# Responses of the picoprasinophyte *Micromonas commoda* to light and ultraviolet stress

**DOI:** 10.1371/journal.pone.0172135

**Published:** 2017-03-09

**Authors:** Marie L. Cuvelier, Jian Guo, Alejandra C. Ortiz, Marijke J. van Baren, Muhammad Akram Tariq, Frédéric Partensky, Alexandra Z. Worden

**Affiliations:** 1 Monterey Bay Aquarium Research Institute (MBARI), Moss Landing, CA, United States of America; 2 Department of Biomolecular Engineering, University of California Santa Cruz, Santa Cruz, CA, United States of America; 3 Sorbonne Universités—UPMC Université Paris 06, CNRS UMR, Station Biologique, CS, Roscoff, France; 4 Department of Ocean Sciences, University of California Santa Cruz, Santa Cruz, CA, United States of America; 5 Integrated Microbial Biodiversity Program, Canadian Institute for Advanced Research, Toronto, Canada; Mount Allison University, CANADA

## Abstract

*Micromonas* is a unicellular marine green alga that thrives from tropical to polar ecosystems. We investigated the growth and cellular characteristics of acclimated mid-exponential phase *Micromonas commoda* RCC299 over multiple light levels and over the diel cycle (14:10 hour light:dark). We also exposed the light:dark acclimated *M*. *commoda* to experimental shifts from moderate to high light (HL), and to HL plus ultraviolet radiation (HL+UV), 4.5 hours into the light period. Cellular responses of this prasinophyte were quantified by flow cytometry and changes in gene expression by qPCR and RNA-seq. While proxies for chlorophyll *a* content and cell size exhibited similar diel variations in HL and controls, with progressive increases during day and decreases at night, both parameters sharply decreased after the HL+UV shift. Two distinct transcriptional responses were observed among chloroplast genes in the light shift experiments: i) expression of transcription and translation-related genes decreased over the time course, and this transition occurred earlier in treatments than controls; ii) expression of several photosystem I and II genes increased in HL relative to controls, as did the growth rate within the same diel period. However, expression of these genes decreased in HL+UV, likely as a photoprotective mechanism. RNA-seq also revealed two genes in the chloroplast genome, *ycf2-like* and *ycf1-like*, that had not previously been reported. The latter encodes the second largest chloroplast protein in *Micromonas* and has weak homology to plant Ycf1, an essential component of the plant protein translocon. Analysis of several nuclear genes showed that the expression of *LHCSR2*, which is involved in non-photochemical quenching, and five light-harvesting-like genes, increased 30 to >50-fold in HL+UV, but was largely unchanged in HL and controls. Under HL alone, a gene encoding a novel nitrite reductase fusion protein (NIRFU) increased, possibly reflecting enhanced N-assimilation under the 625 μmol photons m^-2^ s^-1^ supplied in the HL treatment. NIRFU’s domain structure suggests it may have more efficient electron transfer than plant NIR proteins. Our analyses indicate that *Micromonas* can readily respond to abrupt environmental changes, such that strong photoinhibition was provoked by combined exposure to HL and UV, but a ca. 6-fold increase in light was stimulatory.

## Introduction

Plants and algae alike encounter a wide range of light conditions in nature. Damage induced by high levels of visible spectrum light (HL) and ultraviolet (UV) radiation can cause photoinhibition which is manifested by decreased photosynthetic capacity. Therefore, plants and algae have developed various photoprotection and acclimation mechanisms to reduce damage by HL and UV radiation as well as oxidative damage caused by reactive oxygen species generated during photosynthesis [1–3]. Photoprotective proteins are often coupled with chlorophyll *a/b-* binding light-harvesting complex (LHC) proteins, which collect the photon energy needed for photosynthesis. While LHCs and several classes of photoprotective proteins are nucleus-encoded and targeted to the chloroplast by a transit peptide, many other components of the photosynthetic machinery are encoded by genes in the chloroplast genome of photosynthetic eukaryotes [4]. This machinery has strong similarities in chlorophyte algae, streptophytes (e.g., land plants), and prasinophyte algae, which together form the Viridiplantae [5,6]. Most of its components have been characterized in model chlorophyte algae and plants, such as *Chlamydomonas reinhardtii* and *Arabidopsis thaliana*, respectively.

Several classes of proteins that are structurally related to LHCs appear to be involved in stress responses or photoprotection. For example, the early light-inducible proteins (ELIPs) family [7] is thought to have photoprotective roles [1,8,9]. They were first found to be produced in etiolated pea seedlings during the early phase of greening [10] and a transient increase in expression was also reported during plant chloroplast maturation [11]. ELIPs are encoded on the nuclear genome and have three transmembrane domains like the major LHCs. ELIPs are thought to transiently bind excess chlorophyll released under light stress as a photoprotective mechanism [8,12,13]. Another type of nucleus-encoded stress-response proteins that have homology to chlorophyll *a/b*-binding proteins are LHC Stress-Related (LHCSR) proteins [14–16]. These are present in green algae and non-vascular plants, and, similar to *ELIPs*, *LHCSR* transcripts appear to accumulate under conditions that cause photo-oxidative stress, such as excessive light, as well as CO_2_ deprivation, sulfur and iron deprivation [17–21]. In *C*. *reinhardtii*, LHCSR proteins are responsible for thermal dissipation of excess energy (i.e., non-photochemical quenching, NPQ) by binding and de-exciting photopigments, including chlorophyll [16], and mutants with two of the three *LHCSR* genes deleted do not survive shifts to HL [14]. Orthologs of this gene are present in many photosynthetic eukaryotes but appear to be absent from vascular plants and red algae [14,15,22]. In the former, PSBS seems to play a role similar to LHCSR [23].

For marine algae, light fields vary dramatically as a function of season, depth, load of suspended or dissolved organic material, and latitude. Penetration of UV radiation also depends on multiple factors, including seawater characteristics and geographic location [24]. UV-B (280–320 nm) wavelengths are absorbed more rapidly than UV-A (320–400 nm) but can be prevalent in the upper photic layer. For example, in the Sargasso Sea at 20 m depth, 10% of incident surface UV-B is still present, while UV-B is effectively absent by 70 cm below the surface in the more organic material rich Baltic Sea [25]. Prasinophytes are a group of marine algae that are widespread in marine systems [26–28]. Class II prasinophytes (the Mamiellophyceae) harbor several genera that are picoplanktonic (≤2 μm cell diameter) and represent the smallest photosynthetic eukaryotes. Picoplanktonic members of the Mamiellophyceae are found in environments ranging from the coast to open-ocean [29–31]. Some, like *Micromonas* and *Bathycoccus*, are present from tropical to polar regions, while others (e.g., *Ostreococcus*) have yet to be reported in high-latitude environments. These three genera are thus far the only genome-sequenced representatives of prasinophytes [32–34].

While the structure of the core photosystem I (PSI) and photosystem II (PSII) and of LHCI appears to be globally similar between prasinophytes, chlorophytes and plants, the Mamiellophyceae lack a classical LHCII [22,34,35]. Instead, they possess a unique LHC type, named LHCP (“P” for prasinophyte), which is likely associated with PSII. The phylogenetic position of LHCP proteins outside the clade containing the LHCII polypeptides of plants and chlorophytes make the Mamiellophyceae interesting models for understanding ancestral developments in the Viridiplantae [22,34]. Additionally, the genome sequences of *Micromonas commoda* and *Micromonas pusilla* show that both species have ELIP and LHCSR proteins, as well as another type of putatively photoprotective proteins, one-helix-proteins (OHPs) [13,34]. However, responses of prasinophyte photosynthetic and photoprotective genes to light-shifts and UV-stress have not been characterized and the present study on *M*. *commoda* (RCC299) is the first such analysis. *M*. *commoda* is a representative of Clade A, one of the seven known *Micromonas* clades, and is closely related to Clades B and C but evolutionarily distant from *M*. *pusilla*, which belongs to Clade D [6,30]. The *Micromonas* ABC-lineage as a whole appears to be broadly distributed in temperate and lower latitude oceans, but not high latitude environments where continuous light occurs during summer [6,30,36]. Here, light:dark synchronized *M*. *commoda* RCC299 cultures were investigated over a range of visible light levels. They were also subjected to environmentally-relevant HL or to HL+UV shifts for which cellular and transcriptional responses were investigated using flow cytometry, RNA-seq and qPCR. Our results indicate that this species copes well with HL shifts, but is sensitive to UV radiation over the duration and intensities tested here. Our study provides new insights into the phenotypic plasticity of this ecologically important microbe with respect to light.

## Materials and methods

### *Micromonas* diel and light-shift experiments

An axenic clonal derivative of *M*. *commoda* (RCC299, deposited at the NCMA as CCMP2709) was grown in semi-continuous batch cultures at 21°C on a 14:10 L:D cycle in K medium [37] prepared with artificial seawater (see http://www.mbari.org/phyto-genome/Resources.html). Cultures were diluted daily with fresh medium (or less frequently depending on cell density) so that concentrations never exceeded 6 x 10^6^ ml^-1^ and maintained mid-exponential growth. Cultures were regularly inoculated into organic carbon rich test medium to verify axenicity. The relationship between irradiance and growth was studied in biological triplicates grown in 50 mL glass test tubes at ~6, 55, 150, 240, 350, 470, 620, 750 μmol photons m^-2^ s^-1^ photosynthetically active radiation (PAR) as measured using a QSL-2101 light meter (Biospherical Instruments, San Diego, CA, USA). Low light levels were obtained using neutral density filters (Lee Filters, Burbank, CA, USA). Bulk fluorescence was measured daily at the same morning time point using a 10-AU fluorometer (Turner Design, Sunnyvale, CA, USA). Cells were acclimated to each light level for ≥10 generations and average growth rates were calculated over 3 to 5 consecutive transfers after acclimation by following changes in fluorescence. The latter reflect changes in cell abundance, if measured at the same time point each day in synchronized cultures. At the ~350 μmol photons m^-2^ s^-1^ light level data from only one transfer was available (therefore the mean and s.d. were computed from the three biological replicates) and data from one biological replicate at 6 μmol photons m^-2^ s^-1^ was from a single transfer. Once average growth rates were determined, diel changes in cellular characteristics were studied for cultures grown at three selected irradiances with 2 h interval sampling starting at 6 a.m. (the onset of the light period) for 20 h and 4 h interval sampling for an additional 12 h. Samples were preserved for analysis by flow cytometry (FCM) by adding glutaraldehyde (Tousimis, Rockville, MD, USA, final concentration 0.25%) for 20 min at room temperature in the dark, and subsequently frozen in liquid nitrogen.

For light-shift experiments, axenic RCC299 was acclimated to 100 μmol photons m^-2^ s^-1^ PAR on a 14:10 hour L:D cycle and monitored daily by FCM. Cells were maintained in mid-exponential phase for ≥9 generations before the experiment start by diluting cultures daily with fresh medium to keep a concentration of 2 x 10^6^ cells mL^-1^. The HL and HL+UV exposure experiments were initiated using the above mid-exponential culture, but performed at separate times due to the volumes required for RNA sequencing and associated incubator space limitations. Controls were performed alongside each experiment. For all light-shift experiments, on day one, 1 L glass (controls and HL treatment) or quartz (HL+UV treatment) Erlenmeyer flasks were filled with culture within 30 min before the start time, Time zero (T_0_), which in all cases occurred 4.5 h after lights-on (thus always at the same point in the diel cycle). At T_0_, quadruplicate controls were continued in the same conditions while the quadruplicated treatments were placed into HL (625 ± 35 μmol photons m^-2^ s^-1^), or the same HL level and UV radiation, with an expansion of flasks numbers to accommodate large volume sampling at each time point for shorter term light shift experiments as detailed below. UV was supplied by one UVB-313 (0.7 W m^-2^ at its 313 nm peak) and one UVA-340 (0.75 W m^-2^ at its 340 nm peak) fluorescent tube (Q-Panel Lab Products, Cleveland, USA). Treatment flasks were shaken at ~150 rpm and control flasks were shaken at each time point upon removal from a Percival Incubator. FCM samples were taken from each flask at 0 h (T_0_), 2.5 h (T_2.5_), 6 h (T_6_), 9.5 h (T_9.5_), and 19.5 h (T_19.5_, which includes the 10 hour dark period) for longer experiments. The shorter time course experiments (2.5 h length) were performed separately from the longer term light shift experiments and the flasks were sampled at 0 h (T_0_), 1 h (T_1_) and 2.5 h (T_2.5_) for RNA and FCM (as follows). Because the shorter term light shift experiments were designed to generate enough material for RNA-seq (1 L of culture) four flasks were sacrificed for RNA sampling at each time point. FCM was sampled throughout from all flasks such that for each treatment and control FCM came from 12 flasks at T_0_, from 8 flasks at T_1_, and from four flasks at T_2.5_.

### Flow cytometry

Cultures were monitored live on a daily basis using an Epics XL (Beckman Coulter, Brea, CA, USA) with data acquisition triggered by side scatter (SSC). For the light-shift experiments and the higher resolution diel studies, samples were also analyzed using an Influx (BD Biosciences, Franklin Lakes, NJ, USA) equipped with a 488 nm laser (200 mW output), a 70 μm diameter nozzle and run at 25 μl min^-1^. FALS, SSC and red autofluorescence derived from chlorophyll (692 ± 40 nm band-pass) were recorded for >1 x 10^4^ cells per sample. *Micromonas* and yellow-green 0.75 μm polystyrene beads (Polysciences Inc., Warrington, PA, USA; added as normalization standards) were defined based on fluorescence and SSC (Epics XL) or FALS (Influx) characteristics. Fluorescence and scatter properties of cells were normalized to beads (i.e., all numerical values are in bead relative units). T-tests were performed in SigmaStat (Systat Software, San Jose, CA) to compare growth rates. One way ANOVAs were used to test light intensity effects on normalized mean FALS cell^-1^ and red fluorescence cell^-1^ at each time point (also implemented in SigmaStat).

### RNA sampling, extraction and sequencing

At each RNA time point four 1 L flasks were sacrificed and harvested by centrifugation at 21°C to avoid cold shock; cell pellets were cryo-frozen and then stored at -80°C. RNA was extracted from three of the four biological replicates harvested at each time point, resulting in a total of 24 samples (biological triplicates from: Control T_0_, T_1_, T_2.5_; HL T_1_, T_2.5_; HL+UV T_1_, T_2.5_ and an additional set of T_0_ control samples from the HL+UV experiment). Total RNA was extracted with the RNeasy kit after homogenization using the QIAshredder kit (Qiagen, Germantown, MD, USA) according to the manufacturer’s instructions. 50 μL of extracted RNA was treated with 1 μL of DNase (TURBO DNA-*free*™ Kit, Ambion, Austin, TX, USA) at 37°C for 30 min according to the manufacturer’s protocol. For further purification, a volume of 4.8 M LiCl (LiCl:sample 1:1, v/v) was added, samples were placed at -20°C for 4 to 6 h, and centrifuged at 16,100 x *g* at 4°C for 30 min and then transferred to fresh tubes to which 400 μL 70% ethanol was added. After 10 min on ice, samples were spun at 18,000 x *g* for 5 min. The last two steps were repeated, the supernatant removed and the pellet resuspended in TE buffer. RNA integrity was determined using a 2100 Bioanalyzer (Agilent Technologies, Santa Clara, CA, USA) and total RNA was quantified on the Nano Drop system (Thermo Scientific, Waltham, MA, USA).

For short read sequencing, double stranded (ds) cDNA was synthesized using the Ovation RNA-seq System (NuGEN Technologies Inc., San Carlos, CA) according to manufacturer's instructions except half the recommended amount of first-strand primer mix was used. Briefly, RNA was reverse-transcribed to generate first-strand cDNA using oligo-dT and random hexamer primers. Ds-cDNA was generated using DNA polymerase, and linearly amplified with the Ribo-SPIA method [38]. For all samples, 1 μg of the resulting ds-cDNA was used for library preparation with half of the cDNA having been fragmented by sonication. The complete sample (fragmented and unfragmented cDNA) was electrophoresed on 2.5% agarose gel and size selected for 100–200 bp. After elution, cDNA fragments were blunt-ended and ligated to platform specific ds-bar-coded adapters (New England Biolabs, Ipswich, MA). Bar-coded sets of 12 cDNA libraries were mixed in equal amounts and sequenced on the SOLiD platform (Applied Biosystems, Foster City, CA).

### Transcriptome analyses

Reads were aligned to the RCC299 nuclear genome using BWA [39] and to the plastid genome using Bowtie [40]. The final mapped read numbers were on average 4.08 ± 0.97 million reads per sample, with approximately half of these being aligned to the 73 kb chloroplast genome. For chloroplast genes, reads aligning between start and end coordinates were assigned to that gene. Prior to assignment, chloroplast gene models were improved (primarily by CDS extension) using the transcript data. Extended gene models and those that were unchanged are available at http://www.mbari.org/resources-worden-lab/ and two new protein-encoding genes identified here were deposited in GenBank under accessions KX172140 (*ycf2*) and KX172139 (*ycf1*). For nuclear genes, most reads aligned to the 3′ terminus, within 8 read lengths of the end of existing annotations (see Results and Discussion). Therefore, transcript abundances were based on reads aligned to the last 403 nucleotides of terminal exons after careful annotation of 3′ UTRs based on directionally cloned-Sanger ESTs. Nuclear genes for which the 3′ UTR formed a convergent overlapping pair (with a gene on the opposite strand) were excluded because gene-specific assignments were not possible for the non-directional 3′ biased transcript reads generated here.

Because of the disparity of expression data recovered from chloroplast and nuclear genes (see Results), read counts were normalized to housekeeping genes, specifically the 23S rRNA gene and chloroplast targeted *GAPDH* (XP_002503470), respectively, which showed less variation across the treatments than other potential housekeeping genes (e.g. *Actin*). We analyzed protein coding chloroplast genes as well as nuclear-encoded PSI- (all) and PSII-related antennae proteins, putative photoprotection-related proteins and two heat shock protein genes (*HSP90*.*1* and *HSP90*.*2*). To avoid overestimating fold changes due to low nuclear genome read counts, we required that ≥100 reads map to the respective protein-encoding gene in one or more samples. Thus, a number of nuclear genes were excluded from differential expression analyses due to: i.) reads assigned, if any, were below this minimum cutoff (*LHCSR1*, *LHCSR2*, *ELIP1*, *ELIP2*, *ELIP4* and *ELIP6*) or ii.) they overlapped with other genes in the 3′ region confounding analyses of non-directional RNA-seq data (*LHCP4*, *ELIP5*, and *ELIP3/ PSBS*-like). Additionally, *LHCP2*.*1* and *LHCP2*.*2* are identical in the region where RNA-seq reads mapped and therefore data presented for *LHCP2*.*2* also represents fold-changes for *LHCP2*.*1* (using the present dataset). To determine significance of differential expression analyses an ANOVA was performed on expression data meeting the read cutoff criterion using the R programming language [41]. Data from HL and HL+UV treatments were compared to T_0_ and, separately, to controls from the corresponding time-point. For controls, time-points were also compared two ways, to T_0_ and to the immediately preceding time point (T_0_ for T_1_ and T_1_ for T_2.5_). The T_0_ samples from the two control experiments (associated with either the HL or the HL+UV shift experiments, respectively) were also compared to each other. Only one gene (*psbT*) exhibited a significant difference between these controls (S1 Table).

### Quantitative polymerase chain reaction (qPCR) analyses

For qPCR, single stranded (ss) cDNA was synthesized using the SuperScript® III First-Strand Synthesis System (Invitrogen, Carlsbad, CA, USA) according to the manufacturer’s instructions. Oligo-dT primers and 6 μL total RNA (12.5 ng μL^-1^) were used in total reaction volumes of 60 μL. To check for contaminating genomic DNA, negative control reactions (RT- controls) were also prepared by omitting the reverse transcriptase enzyme.

TaqMan primer-probe sets were designed for three genes (Table 1) using Primer Express software 3.0 (Applied Biosystems, Brea, CA, USA). The primer-probe sequences were compared to the *M*. *commoda* RCC299 genome [34] to confirm that only the genes of interest were targeted. Probes were labeled with the fluorescent reporter FAM (6-carboxyfluorescein) and a non-fluorescent quencher at the 5′ and 3′ ends, respectively. The endogenous control was a previously published MGB™ labeled actin probe [42] with a fluorescent reporter FAM (6-carboxyfluorescein) at the 5′ end and a 3′ non-fluorescent quencher (Table 1).

**Table 1:** Primer/probe set sequences designed and used here for three nucleus genes targeted to the chloroplast and the house-keeping gene *Actin*.

Gene	Accession	Primer/Probe	Sequence 5'-3'
*LHCP1*	XP_002507256	Forward	CGGAGCTTGAGTTGTCAGTTACTC
		Reverse	TCCAGCTTCGGCAAAACC
		Probe	CGGCGGTCGCTTTGACCCC
*LHCSR2*	XP_002506555	Forward	GCGACCACCGGCAACA
		Reverse	GACTTGACAGCCTCCTTGATGTC
		Probe	CAAGATCCAGCCCGGCAAGAAGTACG
*OHP2*	XP_002502054	Forward	TCCTCGTGGGCATGATGAC
		Reverse	ACGGAGATGGTGAGCTTGATCT
		Probe	CCACCGGCGTGGACTTCATCG
*Actin*	XP_002503091	Forward	GCCCTCGTGTGCGATAAC
		Reverse	CCGACGATGGAGGGAAAGAC
		Probe	CCGGCCTTGACCATGC

qPCR was performed using a 7500 Real Time PCR System (Applied Biosystems, Foster City, CA, USA) in reaction volumes of 25 μL with 1x Taqman Gene Expression Master Mix (Applied Biosystems, Foster City, CA, USA), 250 nM probe, 900 nM primers (final concentrations) and 2 μL (175 pg μl^-1^) of cDNA. Cycling parameters were 1 cycle of 50°C for 2 min; 1 cycle of 95°C for 10 min and 40 cycles of 95°C for 15 s followed by 60°C for 1 min. Primer amplicon size was checked by running the qPCR product on 3% agarose gel with a 50 bp Mini ladder (Fisher Scientific, Pittsburgh, PA, USA). The linear dynamic range for each primer-probe set was tested using cDNA prepared from control samples in a serial dilution of RNA. The concentration of RNA added to the cDNA reaction fell within the linear part of the curve, equivalent to a 1:1 conversion between RNA and cDNA. The efficiencies of primer-probe sets were determined using a dilution series of 1) qPCR product (purified with the MinElute PCR purification kit, Qiagen, Germantown, MD, USA) and 2) cDNA. C_T_ values were generated for treatment and control experiments and data were analyzed using the 2^-ΔΔC^_T_ method [43] performed using the 7500 System SDS Software v1.4 (Applied Biosystems, Foster City, CA, USA) with T_0_ as the calibrator. *Actin* (XP_002503091) was selected as the endogenous control because, using qPCR across the treatments herein, it showed less change in C_T_ than *GAPDH* (by qPCR; in RNA-seq data from these experiments *Actin* showed more variability across treatments than *GAPDH*). Statistical significance was evaluated using an ANOVA implemented in SigmaStat.

## Results

### Growth and cellular changes in response to light and the diel cycle

**Fig 1:**
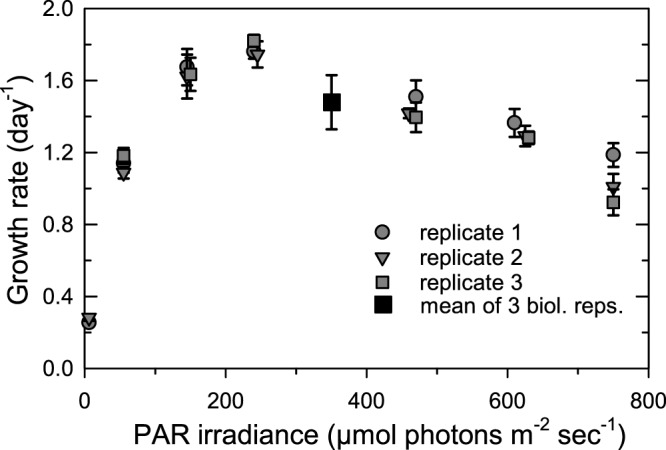
Growth rate as a function of photosynthetically active radiation (PAR) for light-acclimated, mid-exponential phase cultures of *Micromonas commoda* (RCC299). Cultures were grown in biological triplicate on a 14:10 L:D cycle. Symbols represent the mean of 3 to 5 transfers starting ≥10 generations after acclimation to the light level and error bars represent the standard error, except in two cases. For these two, data from multiple transfers post-acclimation were not available, therefore one (at 6 μmol photons m^-2^ s^-1^) has no error represented and the other (350 ± 13 μmol photons m^-2^ s^-1^) shows the mean and standard deviation of the biological triplicates. At the maximum light level tested, 750 μmol photons m^-2^ s^-1^, the μ (0.96 ± 0.04 d^-1^) was significantly lower than at 240 μmol photons m^-2^ s^-1^ where μ_max_ occurred.

**Fig 2:**
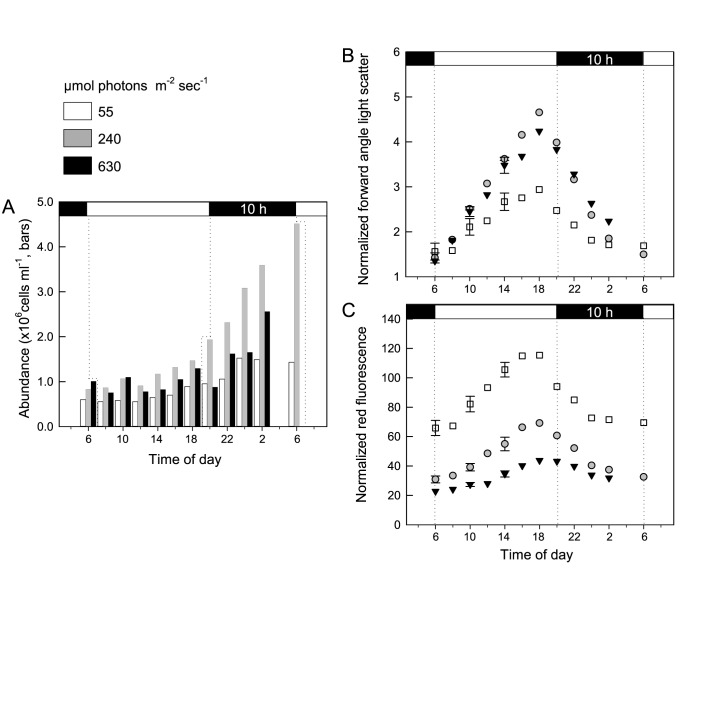
Cellular characteristics over the diel cycle for cultures acclimated to three light levels. (A) Cell abundance, (B) mean FALS in bead relative units (i.e., normalized to beads), and (C) mean chlorophyll-derived red fluorescence in bead relative units. The light (white) and dark (black) periods are indicated in horizontal bars at the top of the graph (14:10 h L:D cycle) and the x-axis is labeled in time of day, with 6 a.m. being lights on. *M*. *commoda* growth rates were 1.10 d^-1^, 1.76 d^-1^ and 1.12 d^-1^ at 55, 240 and 630 μmol photons m^-2^ s^-1^, respectively. These growth rates were similar to those of biological triplicates shown in Fig 1, specifically, 1.04 ± 0.04 d^-1^, 1.78 ± 0.03 d^-1^, and 1.22 ± 0.02 d^-1^, respectively, for similar light levels. Values and error bars for the 6, 10 and 14 h time points in (B, C) reflect the mean and standard deviation of measurements taken during the first diel period and those taken every 4 h until 2 p.m. of the following diel period. Because cell numbers increased with the passage of time, error was not computed from cell abundances in the first and (partial) second diel periods. Note that the HL sample for 6 a.m. on day two was lost due to instrument issues.

*M*. *commoda* grew at ≥1.0 d^-1^ (≥1.4 divisions per day) between 50 to 750 μmol photons m^-2^ s^-1^. Growth rates were significantly slower at 6 μmol photons m^-2^ s^-1^ (μ, 0.28 ± 0.01 d^-1^) and the maximum growth rate (μ_max_, 1.78 ± 0.03 d^-1^) occurred at 240 μmol photons m^-2^ s^-1^ (Fig 1). Cellular characteristics were tightly synchronized with the L:D cycle (Fig 2). Normalized mean FALS cell^-1^ increased throughout the light period (6 a.m. to 8 p.m.), indicating increasing cell size, and started to decrease at the light-to-dark transition, coincident with increased cell numbers (reflecting cell division) at all three light levels tested (Fig 2A and 2B). Like FALS, normalized mean red fluorescence cell^-1^ (representing chlorophyll-derived fluorescence) increased throughout the light period and decreased during night (Fig 2C). The overall amplitude of change was positively related to the culture growth rate for FALS (Fig 2B) and inversely related to the irradiance level for red fluorescence (Fig 2C). It must be noted that FALS values were equivalently low for cells grown at all three irradiances at dawn when the division phase ended (Fig 2B). Thus, the low irradiance, slow growing cultures had the smallest overall average cell size (compared to the two faster growing/higher light level cultures) at all time points except the point of the dark-to-light transition, suggesting that whatever the light level, cell size is the same after the division phase for exponentially growing cells.

### Cellular responses to high light and UV radiation

**Fig 3:**
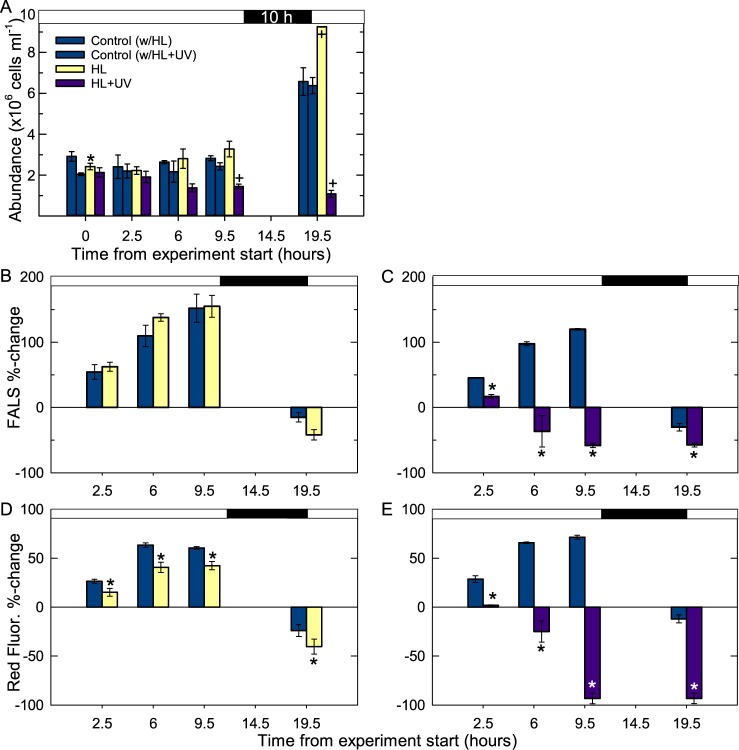
*Micromonas commoda* abundance and cellular characteristics in long-exposure light shift experiments. Cells were in acclimated, mid-exponential growth at 100 μmol photons m^-2^ s^-1^ on a 14:10 L:D cycle for ≥9 generations prior to the shift to 625 ± 35 (SD) μmol photons m^-2^ s^-1^ at the first time point of the experiment, T_0_, which occurred 4.5 h after lights-on. (A) Cell abundance, with light (white) and dark (black) periods indicated by the horizontal bar along the top of graph, treatments and controls are colored as indicated on graph. (B-C) Percent change in bead normalized mean FALS cell^-1^ from the T_0_ value after a shift to (B) HL and (C) HL+UV treatments, as well as the respective controls. Vertical bar coloring is as indicated in (A). (D-E) Percent change in bead normalized mean red fluorescence cell^-1^ in the (D) HL and (E) HL+UV treatments and their respective controls. Cells at T_0_ were 4.5 h into the light period, where red fluorescence and FALS is higher than at lights-on (see Fig 2), hence the negative percent changes at T_19.5_ (the point of lights-on/the end of the dark period) are expected. Data represent the average of biological triplicates and error bars represent the standard deviation. *P*-values <0.05 (*) and <0.001 (+, Fig 3A only) are indicated for significant differences from controls at the same time point (HL vs. control 1, HL+UV vs. control 2).

**Fig 4:**
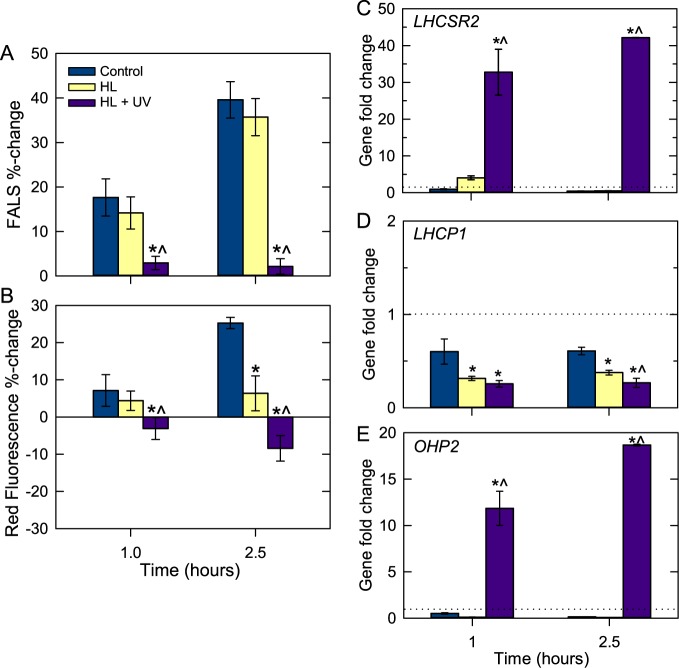
Cellular characteristics of *M*. *commoda* during short-exposure light shift experiments and qPCR analysis of three genes. Percent change in (A) mean normalized FALS cell^-1^ and (B) mean bead normalized red fluorescence for controls and the HL and HL+UV treatments. Data represent the percent change relative to T_0_, which occurred 4.5 h after lights-on, and the x-axis units are time from the experiment start (in hours). Cells were grown at 100 μmol photons m^-2^ s^-1^ for ≥9 generations prior to manipulation. On the day of the experiment, cultures were sampled at T_-4.5_, transferred to flasks and placed in their respective treatments at T_0_. Data represent the average of four to twelve flasks as detailed in materials and methods, and error bars represent standard deviations of these flasks. (C-E) Fold-changes in gene expression for select genes by qPCR. Values >1 increased relative to the T_0_ control, while those <1 decreased. Note differences in y-axis scales. Samples that were significantly different (*p*<0.01) from the control (*) and HL+UV samples that were significantly different from HL at the same time point (^) are denoted. Data represent the average of four biological replicates and error bars represent the standard deviation.

Experimental manipulations involved two light treatments. In the first, acclimated mid-exponential growth cells were shifted from a control (100 μmol photons m^-2^ s^-1^) to higher light level (HL, ~600 μmol photons m^-2^ s^-1^) and in the second they were shifted from the control light level to HL+UV. The experiments were performed for two time-periods: long- (19.5 h, Fig 3) and short- (2.5 h, Fig 4) exposure. In both cases, the experiments were initiated 4.5 h into the light period by shifting cultures from the pre-experiment control conditions to the treatment condition or maintaining them at 100 μmol photons m^-2^ s^-1^ (for controls).

In long-exposure experiments, cultures were shifted after 4.5 h at 100 μmol photons m^-2^ s^-1^ to the HL or HL+UV treatment for the remainder of the light period (9.5 h) then entered the dark night period, which lasted 10 h (the same L:D cycle as for cells leading up to the experiment; Fig 3A). At the experiment onset, the growth rate (1.19 ± 0.14 d^-1^) was not different than the value interpolated for 100 μmol photons m^-2^ s^-1^ from Fig 1. Cell abundance increased in the HL treatment more than the control by 19.5 h (Fig 3A), resulting in a significantly higher growth rate (1.37 ± 0.03 d^-1^, *p*<0.05). Thus, within less than 24 h this HL-shifted culture achieved a growth rate similar to the cultures that were acclimated to 610 μmol photons m^-2^ s^-1^ (1.36 ± 0.15 d^-1^; Fig 1). In contrast, in the shift from control to HL+UV, cell numbers decreased relative to the control (Fig 3A), resulting in a ‘negative’ growth rate.

As expected, changes in bead normalized mean FALS cell^-1^ (expressed as % increase or decrease from T_0_) were similar for the long-exposure experimental controls (Fig 3B and 3C) and cells acclimated to the corresponding light-level in the diel study (Fig 2B). FALS cell^-1^ increased during the remaining part of the light period (9.5 h) by between 120–152% (relative to T_0_ FALS) in the controls of the HL and HL+UV experiments and was at a minimum at T_19.5_ (Fig 3B and 3C). Note that with respect to cell cycle stages, the %-changes in FALS at T_19.5_ relative to T_0_ are most similar to calculating the %-change between the 24 hr and 12 hr time points in the diel (Fig 2B). In the HL treatment, FALS also increased over time (Fig 3B) and did not differ significantly from controls. In contrast, FALS cell^-1^ was significantly lower by T_2.5_ in the HL+UV treatment than the control at the same time point and was lower than the T_0_ value by T_6_ (Fig 3C). Similarly, changes in mean red fluorescence cell^-1^ in the controls (Fig 3D and 3E) were of the same amplitude as in the diel study. This parameter also increased in the HL treatment, but less than in the controls (Fig 3D, *p*<0.05). Under HL+UV exposure, mean red fluorescence cell^-1^ decreased rapidly and was lower than controls by T_2.5_ (Fig 3E). As seen for FALS, T_6_ values were lower than T_0_ and continued to decrease throughout the HL+UV experiment, reaching -93 ± 5% of T_0_ at T_19.5_, without apparent recovery during the dark period.

Changes in cellular characteristics were similar for the controls in the short exposure (2.5 h; Fig 4A and 4B) and longer duration (Fig 3B–3E) experiments at the single comparable time point, T_2.5_. Normalized mean FALS cell^-1^ did not change significantly between the control and HL treatment (Fig 4A), whereas HL mean red fluorescence cell^-1^ was significantly lower than in the control by T_2.5_ (Fig 4B, *p*<0.05). In the HL+UV treatment mean FALS and red fluorescence cell^-1^ were significantly lower by 1 h (Fig 4B), indicating they were no longer progressing through the typical increases that occur across the day period as cells move towards division. Notably, there were no significant differences in FALS or red fluorescence cell^-1^ at T_1_ in the HL possibly reflecting the capacity of cells to buffer variations over short exposure, while in HL+UV large adjustments had already occurred.

### Gene model improvement and RNA-seq analyses

RNA-seq was used to assess *M*. *commoda* responses to light shifts and UV exposure during the short-term experiments. Prior to comparing gene expression levels in the different treatments we observed reads mapped to unannotated regions of the chloroplast genome. Using this data we extended open reading frames (ORFs) for *psbE*, *psbF*, and *psbB* and for the 23S ribosomal RNA (*rrl*). We also identified and deposited two previously unrecognized genes, *ycf2-like (ftsH*, *orf1577*) and *ycf1-like* (*orf603)*. The products of these genes are far more diverged from *Ostreococcus* homologs (ORF1260/YP_717220 and ORF537/YP_717209, respectively) than other proteins encoded by chloroplast genes that are shared by *Ostreococcus* and *Micromonas*. For example, the former protein aligned to just 605 of the 1260 amino acids (a.a.) comprising *Ostreococcus tauri* ORF1260/Ycf2/FtsH (with 30% identity) and blastp to NCBI’s non-redundant database only otherwise recovered a *Bathycoccus prasinos* protein (30% a.a. identity). In contrast, the RNA polymerase beta subunits (RpoB) of these two genera share 68% a.a. identity, and the photosystem I P700 chlorophyll *a* apoproteins A1 (PsaA) are 97% identical. Overall, we identified 60 protein-encoding genes, 26 tRNAs and 6 rRNAs (two copies each of the 16S, 5S and 23S genes) in the chloroplast genome (92 total); these numbers include the 58 protein-encoding, 26 tRNA genes and 3 rRNAs reported previously [34].

**Fig 5:**
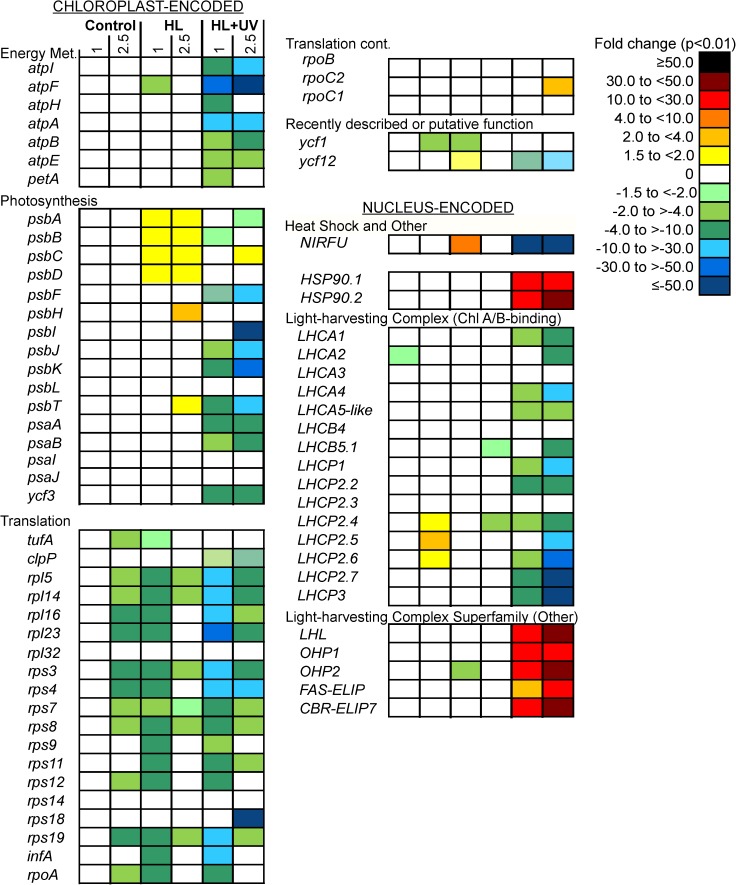
Expression changes for protein-encoding genes from the chloroplast genome and a subset of nuclear genes. HL and HL+UV data represent fold changes relative to controls at the same time point, while each control time point is compared to the preceding control time point. Only genes that met coverage criteria and that displayed significant changes (*p*<0.01) ≥1.5-fold across the biological triplicates in at least one time point, relative to controls at the same time point (this figure), or relative to T_0_ (Fig A in S1 File), are shown. Note that all LHCs and LIL as well as HSP90.2 (XP_002507383) and NIRFU (XP_002507511) proteins have transit peptides targeting them to the chloroplast, as predicted using TargetP.

Approximately half the RNA-seq reads generated mapped to the chloroplast genome (Table A in S1 File). Chloroplast gene transcripts are not poly-adenylated and are recovered well by random hexamers (as used here; Table A in S1 File). Organellar genomes are also typically present in high copy numbers and show elevated levels of expression such that chloroplast transcripts often represent a significant proportion of data generated in RNA-seq experiments involving algae or plants [44]. Additionally, in our study, the reads assigned to nuclear genes generally came from the 3' region, creating issues for analyzing overlapping gene pairs because the reads were non-directional. Due to these biases, expression levels were normalized to housekeeping genes from the respective genome, instead of using standard RNA-seq normalization procedures [45], and a number of nuclear genes of interest had to be excluded from differential expression analysis (see methods). Differential expression patterns were generally similar when treatments were compared to the T_0_ control (Fig A in S1 File) or when they were compared to the control at the same time point (Fig 5, S1 Table). However, the latter allow a more direct assessment of the effects of the treatment, rather than an assessment of the combined effects of diel (as cells progress through the cell cycle) and treatment changes that are represented by comparison to T_0_.

### Expressional responses of protein-encoding chloroplast genes

Fifty-six of 60 protein-encoding genes present in the chloroplast genome were analyzed for expression changes in the short-exposure experiments, while four (*psbN*, *psbZ*, *psaM* and *rpl20*) did not meet the minimum mapped-read number criterion and were excluded from differential analyses. Overall, forty-seven genes showed ≥1.5-fold change (*p*<0.01) relative to the control at the same time point (Fig 5) or to T_0_ (Fig A in S1 File) and nine did not exhibit significant changes (S1 Table).

Chloroplast gene expression between control and HL-shifted cells in the short-exposure treatments exhibited three main patterns (Fig 5, Fig A in S1 File, S1 Table). The first corresponded to genes encoding ATP synthase subunits (*atpA*, *atpB*, *atpE*, *atpF*, *atpH*, *atpI*) and cytochrome *b*_6_*f* complex subunits (*petA*, *petB*, *petG*), which showed no significant changes (*p*<0.01) between controls and HL, except for the ATP synthase CF_0_ subunit B (*atpF*) that showed a moderate under-expression in HL at T_1_. The second pattern involved half the chloroplast genes related to photosystem II (PSII). In HL, genes encoding the PSII reaction center proteins D1 and D2 (*psbA* and *psbD*, respectively) and the PSII core antennae CP43 and CP47 (*psbC* and *psbB*, respectively) showed small but statistically significant increases in expression at both time points, and two other PSII-related genes, *psbH* and *psbT*, also showed modest increases at T_2.5_ (Fig 5). When compared to T_0_, moderate expression increases were observed in the HL treatment for four other PSII-genes (*psbF*, *psbJ*, *psbL*) (Fig A in S1 File). Most other photosystem genes, notably PSI genes, including *ycf3* which encodes the PSI assembly factor Ycf3, did not change significantly under HL relative to both the control at the same time point and to T_0_ (except *psaA* at T_1_ in the latter).

The third pattern, observed in both the controls and HL treatments, involved genes related to transcription and translation machinery (RNA polymerase subunits, SSU-rps (30S) and LSU-rpl (50S) proteins of the chloroplast 70S ribosome, translation initiation factor 1 (*infA*) and elongation factor Tu (*tufA*). For a large number of these genes expression levels decreased significantly from T_1_ to T_2.5_ (7 hours after the onset of light) in the control (Fig 5). In the HL treatment, expression of RNA polymerase alpha subunit (*rpoA*), many ribosomal transcripts and *infA* decreased by 4 to 10-fold relative to both the control at the same time point (Fig 5) and at T_0_ (Fig A in S1 File).

The HL+UV treatment induced a massive reduction in transcripts from chloroplast genes involved in the PSII and PSI reaction centers as well as other categories. Although generally unchanged in the controls and HL, ATP synthase genes encoding the F_0_ and F_1_ subunits were up to 50-fold lower in expression under HL+UV. In contrast, neither *petB* nor *petG* changed significantly and *petA* (cytochrome *f*) levels were lower than the control (2–4 fold) only at T_1_ (Fig 5). Transcript levels for many other genes decreased dramatically by T_1_ and T_2.5_ relative to the corresponding controls (e.g., 30 to 50-fold lower; Fig 5, Fig A in S1 File). Interestingly, *psbC* increased slightly after 2.5 h as did the RNA polymerase beta chain (*rpoC2*) in HL+UV, while *psbA*, *psbB* and *psbD* showed no or little change although they had increased in HL (Fig 5). Other exceptions were for *tufA* which did not change significantly in HL+UV. Finally, although the Rubisco large subunit (*rbcL*) showed expression, no changes were observed in controls or any treatments.

### Nuclear-genome gene expression by RNA-seq

We also analyzed genes involved in the photosynthetic antennae (chlorophyll *a*/*b* binding proteins) and photoprotection. Several LHCA and LHCP proteins, two OHPs, one LIL (*LHL*) and two ELIPs that are encoded on the nuclear genome, and targeted to the chloroplast by a transit peptide, could be analyzed. Of these, changes in LHC and LIL gene expression were minor in the controls and the HL treatment. Just three (*LHCP2*.*4*, *LHCP2*.*5*, *LHCP2*.*6*) of the 15 *LHC* genes evaluated increased significantly between T_1_ and T_2.5_ in the control, exhibiting fold changes between 1.5 to 4 fold (Fig 5, S1 Table). In the HL treatment, expression of most genes was not different from controls at either time point, except for a slight decrease in the one-helix-protein 2 gene (*OHP2*) at 1 h as well as *LHCB5*.*1* and *LHCP2*.*4* at 2.5 h (Fig 5). This indicates that at T_2.5_ in HL *LHCP2*.*5* and *LHCP2*.*6* had increased to the same extent as the diel-related changes observed in the control. Different from the HL shift, the HL+UV shift caused a significant decrease in the expression of most LHC genes relative to the control, with a 4 to 50-fold drop by the 2.5 h time point (Fig 5). In contrast, *LHL*, *FAS-ELIP* and *CBR-ELIP*, the two *OHPs*, as well as two genes coding heat shock proteins, *HSP90*.*1* and *HSP90*.*2*, were all highly expressed in HL+UV relative to both the control at the same time point (Fig 5) and at T_0_ (Fig A in S1 File).

Lastly, a gene coding a putative ferredoxin-nitrite reductase protein in *M*. *commoda* (named here NIRFU, XP_002507511) showed increased expression under HL but decreased dramatically in HL+UV relative to the controls at the same time point and at T_0_ (Fig 5). This nuclear gene-encoded protein has a predicted chloroplast transit peptide at the N-terminus and showed homology to ferredoxin-nitrite reductases of plants [46], e.g. 53% identity to NIR in *Solanum lycopersicum*. However, *M*. *commoda’s* NIRFU also contains a C-terminal rubredoxin (a non-heme iron binding domain with an iron-sulfur center known to be involved in electron transfer) and a domain belonging to the Ferredoxin NADP^+^ reductase superfamily.

### Gene expression by qPCR

We developed qPCR primer-probe sets for three nuclear *LHC/LIL*-related genes, including one of two *LHCSR* genes in *M*. *commoda*. The product of the *LHCSR2* (XP_002506555) gene assayed here shows 63% amino acid identity with *LHCSR1* (XP_002501722). By comparison, identity between the three *Chlamydomonas LHCSR* homologs is 82% and 100% (two are identical copies). *LHCSR* genes did not meet the mapped read number criterion for RNA-seq differential expression analysis and by qPCR less than 1.5-fold changes were observed for *LHCSR2* in the controls and HL, except for HL 1 h (Fig 4C). The expression levels did not differ significantly between controls and HL at either time point. Under HL+UV, *LHCSR2* expression increased 33-fold (T_1_) and 42-fold (T_2.5_) relative to T_0_ and was significantly different (*p*<0.01) than control and HL treatments at the same time point (Fig 4C).

The other two nuclear genes analyzed by qPCR did meet the mapped read number criterion for RNA-seq. Different housekeeping genes and normalization methods were used for qPCR [43] and RNA-seq analyses (see methods), making trends comparable, but not exact fold changes. By qPCR, *LHCP1* expression in the control showed minimal (-1.7, T_1_; -1.6, T_2.5_ when converted from decimal values) fold changes from T_0_ and the most comparable RNA-seq analysis did not exhibit significant changes (Fig A in S1 File). In HL, -2.7 to -3.2-fold changes from T_0_ were observed by qPCR, and expression was lower than the controls at the same time point, but again changes were not visible in the RNA-seq analysis. The largest changes occurred under HL+UV (Fig 4D), with -3.9 (T_1_) and -3.7 (T_2.5_) fold changes from T_0_ by qPCR, and -3.1-fold (T_1_) and -7.0-fold (T_2.5_) by RNA-seq relative to T_0_ (Fig A in S1 File, S1 Table). Under HL+UV, *LHCP1* expression was significantly lower than controls for both data types. For *OHP2*, the magnitude of response was much greater than *LHCP1* (Fig 4D and 4E). Trends were the same for *OHP2* by qPCR and RNA-seq in the HL, where expression decreased relative to T_0_. QPCR again showed larger decreases than RNA-seq and also showed a moderate negative fold-change in the control. *OHP2* expression increased dramatically under HL+UV, and the qPCR (Fig 4E) and RNA-seq data were within the same range at both time points (RNA-seq category 10 to <30-fc; Fig A in S1 File). Because *OHP2* expression declined under the shift to HL (and in the control), the massive induction of *OHP2* in the HL+UV treatment appears to have been triggered by UV exposure.

## Discussion

### Responses to the diurnal cycle and experimental light manipulations

We show that *M*. *commoda* grows under a broad range of light intensities, including irradiances higher than those generally encountered in the upper 40 m of the photic zone where this alga appears to thrive [25,47,48]. *Micromonas commoda-like* strain CCMP489, another Clade A member [6,30,36], and several *Ostreococcus* strains including *O*. *tauri*, reportedly grew slower [49–51] than RCC299 at equivalent light levels. Differences in growth conditions, such as a shorter light period (12 h) and the medium used may however have contributed to offsets between our study and prior research. Indeed, when *O*. *tauri* was grown under the same irradiances and photoperiod as used here, it displayed comparable growth rates as *M*. *commoda*, i.e., 0.29 ± 0.01 d^-1^ at low irradiance (6 μmol photon m^-2^ sec^-1^) and a μ_max_ of 1.74 ± 0.06 d^-1^ [52]. Growth data also exist for other prasinophytes, e.g. [53], and the chlorophyte *C*. *reinhardtii* [14,54]. However, even more extensive differences in culturing conditions make comparisons difficult, urging further comparative studies that use standardized handling, photoperiod and medium.

FALS values have previously been shown to relate to cell size and carbon content in *M*. *commoda-like* strain CCMP489 [51]. Our flow cytometry data on *M*. *commoda* strain RCC299 showed that the maximum normalized mean FALS and chlorophyll-derived red fluorescence cell^-1^ occurred just prior to the end of the light period, regardless of the light level (Fig 2). This indicates that cell size is the same at dawn, regardless of the light level (also likely reflecting cells being in the G_1_ cell cycle phase), but deviates over the remainder of the diel cycle at the different irradiances. Overall differences in chlorophyll-derived red fluorescence in cultures acclimated to different irradiances are consistent with prior work on *C*. *reinhardtii* showing that chlorophyll cell^-1^ is inversely proportional to irradiance level in light-acclimated cells [14,54,55]. The variations we observed over the L:D cycle for red fluorescence and FALS cell^-1^ are similar to those in *M*. *pusilla* CCMP490 [56], *M*. *pusilla* CCMP1545 [57] and *O*. *tauri* [58]. These patterns correlate with cell division and highlight the critical nature of light as a regulator for synchronizing photoautotrophic growth, with timing that varies according to the L:D program.

Exponentially growing *M*. *commoda* cells responded to the HL shift with a rapid increase in growth rate, that was already manifested 19.5 h after the shift by higher cell abundances than in controls (Fig 3A). Mean FALS cell^-1^ increased in a similar way to the control, while mean red fluorescence cell^-1^ was significantly lower (by 11.2 ± 4.6%) within 2.5 h of HL exposure indicating that photoacclimation processes were underway (Fig 3B and 3D). By comparison, continuous-light grown *C*. *reinhardtii* cultures transferred from 75 μmol photons m^-2^ s^-1^ to 750 μmol photons m^-2^ s^-1^ increased in growth rate from 0.87 d^-1^ to 2.22 d^-1^ 8 to 12 h after the shift [59]. In other studies on *Chlamydomonas*, red fluorescence cell^-1^ decreased by 20 to 60%, depending on experiments, during the first 6 h of HL exposure for cultures in continuous light [60,61] and on a L:D cycle [54]. In the latter study, decreased red fluorescence was associated with a decline in chlorophyll content, which started as early as 30 min after HL exposure. Indeed, down-regulation of light-harvesting in response to increased light is the basis of the current model of photoacclimation and growth in phytoplankton [62–65]. This model involves a quick acclimation of metabolic processes, especially growth and photosynthesis, the speed of this process being likely critical for taxa that reside in highly variable aquatic environments.

While the ~6-fold increase of visible light in our HL treatment was beneficial for *M*. *commoda* growth, the concomitant shift to HL plus UV radiation was detrimental. The UV used here included UV-A, which is the more abundant species in natural sunlight, and UV-B which is increasing in some regions due to depletion of the ozone layer [66]. HL+UV had a dramatic effect on both growth and cell characteristics, i.e. FALS and red fluorescence cell^-1^ (Fig 4A and 4B). The spectral output of the UV-A bulb used matches the output levels of natural sunlight between 300 and 340 nm and at higher wavelengths declines to 0 W m^-2^ [67]. The UV-B output of the bulb used was about 3.5 fold-higher than that in natural sunlight. Both UV types will have been reduced from the bulb-output level by seawater attenuation in the experimental quartz flasks as would occur in the surface ocean conditions. Typical wind mixing of ocean surface waters causes similarly rapid shifts as those performed here. However, it is unlikely that UV exposure would be of the duration (and UV-B dose, [25]) studied here, which were designed to trigger physiological and gene expressional changes that could be unambiguously assigned to the HL+UV treatment. *Micromonas* populations that are in the upper photic layer for long periods prior to being shifted even closer to the surface, may be better acclimated for increased UV exposure than our cultures. Indeed, in *C*. *reinhardtii*, pre-acclimation to low levels of UV-B increases survival rates upon a shift to higher UV-B compared to cells that are not pre-acclimated [68]. Collectively, such studies are important for understanding phytoplankton responses to life in increasingly stratified water columns, a predicted outcome of global warming for some oceanic regions [69].

### Expression changes associated with the diel cycle and light shifts

Several studies have addressed prasinophyte growth over the diel cycle with respect to timing of division and cellular characteristics [56–58,70]. Fewer have investigated diel transcriptional changes of chloroplast and nuclear genes [57,71], and to our knowledge none have yet addressed expressional responses in *M*. *commoda* or prasinophyte responses to light shifts. Such studies are important for understanding basic biology of these cells and for future analyses of field metatranscriptome data. Our study followed just 2.5 h of the midday period of L:D synchronized cells, and therefore primarily addresses how cells respond to a rapid shift into HL or HL+UV. Caveats remain on the extent to which transcriptional data reflects protein level data and this affects interpretation of results. For example, *rbcL* expression levels did not change between treatments and controls at the same time point or at T_0_ (S1 Table), and this gene is known to be regulated at the translational level in *C*. *reinhardtii* [72]. More generally, several *Chlamydomonas* studies have not found clear diel changes in chloroplast gene expression [73,74], leading to the proposal that many are under translational control, in sharp contrast with patterns in cyanobacteria where nearly all genes are expressed rhythmically (see e.g. [75,76]). In addition, a comparative proteomic/transcriptomic study of *M*. *pusilla* found a significant number of hypothetically post-transcriptionally regulated (HPTR) genes, although the focus was not on chloroplast genes [77]. Finally, the UV exposure used here was clearly inhibitory to growth of *M*. *commoda*. Thus, it is important to consider that inhibition of growth removes an important carbon and energy sink that could influence the acclimation potential of the cells in our HL+UV treatment. The changes observed in the HL+UV treatment could therefore reflect more indirect effects of the stressor, short-circuiting acclimation mechanisms for accommodating UV stress, rather than direct effects. With these caveats in mind, we discuss the more notable gene expression patterns observed in our study.

### Acclimative changes in genes for photosynthesis and light-harvesting machinery

We investigated a suite of photosynthetic antenna-related proteins, encoded by genes on the nuclear genome, as well as chloroplast encoded genes involved in photosynthesis. A study on *M*. *pusilla* showed that many components of the photosynthetic machinery encoded on the nuclear genome that are associated with the structure of PSII and PSI have clear diurnal expression patterns, with peak expression at the dark-to-light transition [57]. But that study did not investigate changes in *LHC* genes or genes on the chloroplast genome. Additionally, *M*. *pusilla* differs from *M*. *commoda* in having phytochrome, a light sensor regulatory protein, which has been implicated in timing of the dawn expression of photosynthetic machinery [57]. The nuclear genomes of both *M*. *pusilla* and *M*. *commoda* contain genes for chloroplast-targeted PSI-associated LHCs (*LHCA1-4*, *LHCA5-like*), minor polypeptides of PSII (*LHCB4*, *LHCB5*.*1* also known as CP29 and CP26, respectively; CP24 is absent) and prasinophyte-specific LHCs (*LHCP1*, *LHCP2*.*1–2*.*7*, *LHCP3*, *LHCP4*) that are likely analogous to the PSII-associated major LHCB proteins of plants [34,35,78]. Most LHC protein-encoding genes analyzed here did not change significantly in the control or HL treatment apart from small increases associated with the diel cycle in *LHCP2*.*4*, *2*.*5* and *2*.*6* (Fig 5). Exceptions in the HL-shift were for *LHCA1*, *LHCP2*.*5*, and *LHCP3* which showed 2 to 4-fold increases relative to T_0_ 2.5 h after the shift (Fig A in S1 File), suggesting a central role in adjusting photon collection to a stimulatory light-shift. The minimal responses observed for the *LHC* genes may in part be due to the midday period investigated. In *M*. *pusilla*, nucleus-encoded photosynthesis-related genes exhibited highest expression early in the day with lower levels at time points similar to those sampled here (later in the light-period) [57]. Furthermore, as observed here for *LHCB5*.*1*, under HL *Arabidopsis*, *Chlamydomonas* and *Ostreococcus* showed more notable effects on the minor polypeptides of the PSII antenna, which channel energy towards the reaction centers by linking the outer LHCII antennae to reaction centers [22,79,80]. In *Chlamydomonas*, significant transcriptional downregulation has been documented for both *LHCB4* (CP29), e.g. by 75–80% within 1 h of HL exposure, and LHCB5 (CP26) under HL stress [59,61,81,82]. Changes in expression levels of *M*. *commoda* chloroplast photosynthesis genes were also minor in HL relative to controls (Fig 5, Fig A in S1 File).

Chloroplast genes involved in translation responded differently in the control and HL than those directly involved in photosynthetic machinery. No translation-related genes increased in expression in the control along the time course (which started 4.5 h into the light period), and most showed diel-based reductions (Fig 5). The same was observed for the HL treatment but with an earlier onset (Fig 5). Likewise, in *O*. *tauri*, the expression of genes encoding chloroplast ribosomal proteins was shown to be co-regulated during the photoperiod, and to decrease starting about 3 h after the onset of the light period [71]. Furthermore, *O*. *tauri* nuclear- and chloroplast- ribosomal genes were expressed at different times of the day (i.e., the nucleus genes were more highly expressed at the end of the dark period) [71], speaking to the fundamentally different roles these two gene suites have, and to aspects of autonomy retained by the plastid.

Finally, the general lack of change in expression of genes coding for energy metabolism proteins in the HL treatment (only *atpF* declined; Fig 5) indicated that levels of ATP synthase and cytochrome *b*_*6*_*f* per chlorophyll were higher in cultures shifted to HL. In *C*. *reinhardtii*, only slight and non-significant changes were observed after a shift to HL in the relative amount of most proteins coded for by these genes [55]. Moreover, when protein content was normalized to chlorophyll content, cytochrome *b*_6_*f* and ATP synthase over-accumulated in HL [55]. *M*. *commoda* cells in the HL treatment may therefore have had higher capacity in linear electron transport which could have produced the increase in ATP production necessary for facilitating the observed increase in growth rate. Overall, *M*. *commoda* transcriptional responses to the HL treatment were small (Fig 5, Fig A in S1 File) and this is consistent with the 6-fold increase in light intensity being stimulatory, not photoinhibitory. These results suggest that *M*. *commoda* is well adapted for growth under high light levels as well as rapid shifts in irradiance, as commonly encountered in the upper sunlit layer of oceans, and is able to immediately translate light-availability into increased growth rate.

### HL+UV induced responses of photosynthesis and antennae genes

In contrast to results for the controls and HL shift, dramatic reductions were observed for multiple genes in the HL+UV treatment. Decreases in expression of translation-related genes were of a larger magnitude in HL+UV than in the HL treatment or the control. Additionally, in HL+UV most of the six ATP synthase genes showed significantly decreased expression, while the expression of genes coding for cytochrome *b*_6_*f* were little or un-affected. Cytochrome *f* is also considered a stable protein [83–85], potentially explaining the slight decrease of *petA* gene expression with regard to the other two *pet* genes (Fig 5). These results suggest that in *M*. *commoda* cells, UV radiation induces a dramatic reduction of the *de novo* synthesis of ATP synthase but not of the *b*_6_*f* complex, possibly implying that ATP production is more strongly affected by UV. HL+UV also induced an immediate reduction in all major components of the PSII and PSI associated antennae (all *LHCA*, *LHCB* and *LHCP* genes) even though several PSI and PSII associated genes were not strongly effected at the transcriptional level (Fig 5). The latter included *psbA* which codes for the D1 protein (Fig 5) and is considered the most rapidly turned-over chloroplast protein, with even faster turn over than nucleus-encoded LHC proteins [86,87]. Our expression data on *M*. *commoda* suggest that UV did not significantly alter D1 synthesis and repair, but could still strongly affect PSII activity, given the large apparent decrease in chlorophyll-derived red fluorescence cell^-1^. In *C*. *reinhardtii*, UV causes 25% lower D1 protein levels after 1.5 h of exposure and also lower levels (to a lesser extent) of the D2 protein while neither was significantly degraded in controls [88]. PSII activity under UV was also reduced dramatically (by 72%) compared to the control. Reduction in *C*. *reinhardtii* D1 and D2 protein levels are most severe for non-UV acclimated cells. Cells first acclimated to low levels of UV-B show higher levels and faster re-synthesis of D1 and D2 protein, and greater survival rates after being shifted to a higher UV-B dose [68]. Our goal here was to identify for the first time genes involved in the biological responses of *M*. *commoda* to HL+UV. The significant reduction in chlorophyll-derived red fluorescence cell^-1^ that accompanied these expressional changes is suggestive of pigment photobleaching, and *M*. *commoda* did not divide during the experiment, even though UV exposure ceased at the end of the light period. These changes to cell characteristics, growth and transcription are indicative of a massive initial reprogramming to compensate for the UV dose but nevertheless the changes failed to support continued growth.

### Molecular evidence for LHCSR-based NPQ in *Micromonas*

Considerable attention has been given to the LHCSR protein [14–16,55,89]. This protein is responsible for one of the key NPQ mechanisms that allow excess light energy to be dissipated as heat. Although our RNA-seq data did not meet the criteria for differential expression analysis, the qPCR results suggest that *M*. *commoda’s LHCSR2* is triggered similarly to *LHCSR3* in *Chlamydomonas*. We observed a modest, transient increase in *LHCSR2* expression after 1 h and a subsequent reduction at T_2.5_ in HL in *M*. *commoda* (Fig 4*)*. Likewise, in *Chlamydomonas*, *LHCSR3* expression increased within 30 min of transfer to HL and then declined within 1 to 3 h of exposure to HL [20]. *LHCSR* and a number of other photoprotective *LIL* genes (*PSBS1*, *PSBS2*, *ELIP1*, *ELIP5* and *LHL4*) also increased by >10-fold in *C*. *reinhardtii* following 1 h UV-B exposure [68]. Here, HL+UV induced an increase of over 30-fold at T_1_ and 40-fold by T_2.5_ (Fig 5). Our results constitute the first experimental evidence that LHCSR-genes are transcriptionally activated in *Micromonas* by HL and HL+UV. This suggests that LHCSR-mediated NPQ plays a role in accommodating increased light-energy (HL treatment) and photoinhibitory stress (HL+UV) in *M*. *commoda* and likely prasinophytes as a whole.

### Stress-related, lesser known and unique genes exhibiting significant changes

Nuclear encoded LIL proteins in *Micromonas* primarily belong to the OHP and ELIP families, which have different numbers of helices [13,34,78]. In plants and algae, OHPs have one helix while ELIPs have three helices and are thought to have photoprotective functions involving potential binding of chlorophylls released during HL stress [1,13,61]. Multiple *ELIP* genes (six *ELIPs* and one *FAS-ELIP*) are present in the *M*. *commoda* genome, raising the question of possible redundant functions. Here, only *M*. *commoda*’s *CBR*-*ELIP7* and *FAS-ELIP* could be analyzed (see methods). Both proteins have the three helix structures known from most ELIPs [13,34] plus additional domains. They exhibited large significant increases in the shift to HL+UV, but not in the controls or HL. The magnitude of response was lower for the *FAS-ELIP*, that encodes a unique protein found thus far only in the Mamiellophyceae based on available genomic data. It combines the chlorophyll *a/b* binding domain (at the C-terminus, three helices) with a Beta-Ig-H3/fasciclin domain (at the N-terminus, following the predicted transit peptide). Other proteins containing the latter domain are present in *M*. *commoda*, but not in this fused arrangement. In plants and animals, this domain appears to be involved in cell adhesion and development, but a function in single celled organisms has not been described. Neilson and Durnford hypothesized that the domain has been co-opted to facilitate protein-protein interactions or act as part of a signaling pathway [13]. The responses observed here constitute the first report of differential expression of a gene encoding a fusion protein with these domains.

The *LHL* gene of *M*. *commoda* has homology with the *LHL4* gene of *Chlamydomonas* which encodes a three-helix protein whose expression is thought to be mediated by a blue/UV-A light receptor [90]. This LIL protein is present in other chlorophytes and prasinophytes but, unlike ELIPs and OHPs, does not have known homologs in vascular plants [61]. High *M*. *commoda LHL* expression under the shift to HL+UV (Fig 5) corresponds to responses in *C*. *reinhardtii* under UV exposure. We also observed a transient increase in expression relative to T_0_ for the HL treatment. In general, the response of this gene mirrored that of the *ELIPs* and *OHPs* analyzed here in *M*. *commoda*.

OHPs are thought to be more strictly related to light stress than ELIPs [12,91]. Here, the only change observed in the control and HL for the two *Micromonas OHP* genes was a transient slight (but significant) decrease in *OHP2* 1 h into the HL shift. This change was evident in both the RNA-seq (Fig 5) and qPCR (Fig 4E) data. Thus, it appears that the growth stimulatory HL used during our experiments does not induce increased expression of either *OHP* gene. However, in the HL+UV treatment we observed large increases in *OHP1* (in RNA-seq) and *OHP2* (10- to 50-fold within 2.5 h) transcript levels by both RNA-seq and qPCR. In *A*. *thaliana*, homologs of the *M*. *commoda’s* OHP2 are localized in the thylakoid membranes and the accumulation of *OHP2* transcripts (and protein) has so far been associated with HL shifts, since other stresses such as UV-A, heat, cold, desiccation or oxidative stress did not result in expressional changes [92]. Other studies have reported up-regulation of the *Arabidopsis OHP1* gene under HL [93] and in *C*. *reinhardtii OHP1 (LHL2)* was induced within 1 h after a shift from LL to HL, and then decreased to initial levels within 6 h [61]. While cyanobacteria lack ELIPs, they do have one-helix proteins (termed HLIPs), and these *hli* genes increase in expression under both HL and UV-B stress [11,94,95]. Our results indicate that in *M*. *commoda* the induction of *OHP2* is more sensitive to UV radiation than the stimulatory HL used here. Our data supports the proposal that in eukaryotes OHPs are essential for photoprotection against UV radiation, which is also thought to activate specific receptors in plants [92].

Changes were also observed for *M*. *commoda* genes that have recently described or uncharacterized functions. Among chloroplast genes, the protein encoded by *ycf12* (Psb30) has now been demonstrated to serve as a PSII reaction center subunit in *C*. *reinhardtii* [96]. We saw a transient increase in *ycf12* expression when cells were shifted to HL and a large reduction in the HL+UV treatment. These results fit with Ycf12 being required for optimal PSII functioning under HL. The Ycf1 protein of *M*. *commoda* is highly divergent from those of plants, making assignment of function more tentative than for Ycf12. *Ycf1* decreased in expression in association with the diel cycle in the control, and this decrease was accelerated in the HL treatment (transient; Fig 5). These patterns were most similar to those of *tufa*, which codes for an elongation factor (Fig 5). In *A*. *thaliana*, *Ycf1* encodes Tic214, part of the “translocon at the inner envelope membrane of chloroplasts” (TIC) complex that facilitates transiting of proteins encoded on the nuclear genome into the chloroplast [97,98]. TIC is necessary for viability in *A*. *thaliana* and presumably other plants and green algae. If Ycf1 does have the same function in *M*. *commoda*, then the fact that its response is similar to that of an elongation factor further emphasizes the strong diurnal patterns connected to the L:D cycle, timing of translocation to the chloroplast, and resulting synchronized growth in photosynthetic organisms.

Finally, the N-terminal region of the novel NIRFU fusion protein is homologous to plant NIR proteins (nitrite reductase). Six electrons in total are required to reduce nitrite to ammonia which can then be used in downstream steps of the nitrogen assimilation pathway of photosynthetic organisms [99,100]. The electron accumulation in plant NIR occurs in one-electron steps because ferredoxin is a one electron donor and the enzyme contains just one ferredoxin binding site [99,100]. However, the full NIRFU protein contains NIR fused to predicted domains for rubredoxin, which can act as an electron transfer protein [101], and the ferredoxin reductase superfamily domain (FNR, cytochrome *b*_5_ reductase-like). Apart from *M*. *commoda*, this fusion protein only appears to be present in *O*. *tauri*, *B*. *prasinos* and *M*. *pusilla* based on blastp queries against available plant and algal genomes (GenBank nr database). Although the three-dimensional structure and function of NIRFU is not known, some aspects of the combination of domains have similarities to bacterial nitrite reductase operons. The cytochrome *c* nitrite reductase (*nrfA*) operon of several non-photosynthetic bacteria contains not only *nrfA* (which encodes the catalytic subunit), but also *nrfH*, encoding a tetraheme cytochrome *c* [102]. Together, these form a complex that optimizes the efficiency of electron transfer to nitrite, the terminal acceptor [102]. We hypothesize that the additional domains in NIRFU enhance the efficiency of electron transfer over classical plant NIR proteins, potentially with both ferredoxin and NAD(P)H dependent nitrite reduction in the chloroplast. The culture medium used for our *M*. *commoda* experiments had nitrate as the primary nitrogen source. While *NIRFU* expression went down in HL+UV, consistent with a cessation of growth and the associated demand for ammonia, *NIRFU* expression increased dramatically by 1 h in the HL treatment (note that expression of nitrate reductase could not be evaluated). This may indicate that these picoprasinophytes use *NIRFU* to rapidly increase the supply of ammonia to the nitrogen assimilation pathway, fueling enhanced rates of growth under sudden changes in light availability.

## Conclusions

Our results suggest that *M*. *commoda* is well adapted for growth under HL levels encountered in surface waters of aquatic environments. Photoinhibitory processes due to UV may be counteracted in part by increased synthesis of LHCSR2, which exhibited >30-fold higher gene expression, and upregulation of other potential compensatory mechanisms (e.g., products of *HSP* and *LIL* genes) that did not change significantly with HL alone. It appears that *M*. *commoda* responds to HL by transient dissipation of excess energy, then by launching changes in the antennae complex that were manifested in decreased levels of chlorophyll-derived red-fluorescence per cell within 2.5 hours. Despite a six-fold increase in light intensity, no adjustment was apparent at the transcriptional level in the HL treatment relative to controls for chloroplast genes (apart from a few components of PSII, including D1 protein, and translation related proteins), or most antennae related proteins encoded on the nuclear genome. These results may indicate that post-translational controls act on the gene suites that did not exhibit expressional changes, such that the RNA-seq was not reflective of downstream protein modifications. Overall, we observed an immediate capacity of *M*. *commoda* to accommodate the rapid shift in light level, resulting in increased growth rate on the same day as the shift in these diel studies. Although further comparative studies with other taxa are needed, these findings translate to a level of environmental flexibility that may contribute to the success of *M*. *commoda*, and possibly the entire genus, in a variable marine water column.

## Abbreviations

Gene and protein abbreviations are indicated in the following styles: Nuclear encoded proteins: all caps (YCF1); Chloroplast encoded proteins: title case (Ycf1); Nuclear genes: uppercase italic (*YCF1*); Chloroplast genes: lowercase italic (*ycf1*).

## Supporting information

S1 File Supporting Information. This file contains supplementary Fig A and Table A.
(DOCX)

S1 Table *Micromonas commoda* (RCC299) chloroplast genes as well as select chloroplast-targeted and light-harvesting related genes that could be analyzed herein. (XLS)

## References

[1] Hutin C, Nussaume L, Moise N, Moya I, Kloppstech K, Havaux M (2003) Early light-induced proteins protect *Arabidopsis* from photooxidative stress. Proc Natl Acad Sci U S A 100: 4921–4926. 10.1073/pnas.0736939100 12676998 PMC153656

[2] Norén H, Svensson P, Stegmark R, Funk C, Adamska I, Andersson B (2003) Expression of the early light-induced protein but not the PsbS protein is influenced by low temperature and depends on the developmental stage of the plant in field-grown pea cultivars. Plant, Cell & Environment 26: 245–253.

[3] Niyogi KK (1999) Photoprotection revisited: Genetic and molecular approaches. Annu Rev Plant Physiol Plant Mol Biol 50: 333–359. 10.1146/annurev.arplant.50.1.333 15012213

[4] Green BR (2011) Chloroplast genomes of photosynthetic eukaryotes. Plant J 66: 34–44. 10.1111/j.1365-313X.2011.04541.x 21443621

[5] Lewis LA, McCourt RM (2004) Green algae and the origin of land plants. American Journal of Botany 91: 1535–1556. 10.3732/ajb.91.10.1535 21652308

[6] van Baren MJ, Bachy C, Reistetter EN, Purvine SO, Grimwood J, Sudek S, et al (2016) Evidence-based green algal genomics reveals marine diversity and ancestral characteristics of land plants. BMC Genomics 17. 10.1186/s12864-016-2585-6 PMC4815162 27029936

[7] Heddad M, Adamska I (2002) The evolution of light stress proteins in photosynthetic organisms. Comp Funct Genomics 3: 504–510. 10.1002/cfg.221 18629257 PMC2448420

[8] Montane MH, Kloppstech K (2000) The family of light-harvesting-related proteins (LHCs, ELIPs, HLIPs): was the harvesting of light their primary function? Gene 258: 1–8. 11111037 10.1016/s0378-1119(00)00413-3

[9] Heddad M, Noren H, Reiser V, Dunaeva M, Andersson B, Adamska I (2006) Differential expression and localization of early light-induced proteins in *Arabidopsis*. Plant Physiol 142: 75–87. 10.1104/pp.106.081489 16829586 PMC1557597

[10] Meyer G, Kloppstech K (1984) A rapidly light-induced chloroplast protein with a high turnover coded for by pea nuclear DNA. Eur J Biochem 138: 201–207. 6692824 10.1111/j.1432-1033.1984.tb07900.x

[11] Dolganov NA, Bhaya D, Grossman AR (1995) Cyanobacterial protein with similarity to the chlorophyll *a/b* binding proteins of higher plants: evolution and regulation. Proc Natl Acad Sci U S A 92: 636–640. 7831342 10.1073/pnas.92.2.636 PMC42797

[12] Adamska I, Kruse E, Kloppstech K (2001) Stable insertion of the early light-induced proteins into etioplast membranes requires chlorophyll-alpha. Journal of Biological Chemistry 276: 8582–8587. 10.1074/jbc.M010447200 11114311

[13] Neilson JA, Durnford DG (2010) Evolutionary distribution of light-harvesting complex-like proteins in photosynthetic eukaryotes. Genome 53: 68–78. 10.1139/g09-081 20130750

[14] Peers G, Truong TB, Ostendorf E, Busch A, Elrad D, Grossman AR, et al (2009) An ancient light-harvesting protein is critical for the regulation of algal photosynthesis. Nature 462: 518–U215. 10.1038/nature08587 19940928

[15] Bailleul B, Rogato A, de Martino A, Coesel S, Cardol P, Bowler C, et al (2010) An atypical member of the light-harvesting complex stress-related protein family modulates diatom responses to light. Proc Natl Acad Sci U S A 107: 18214–18219. 10.1073/pnas.1007703107 20921421 PMC2964204

[16] Bonente G, Ballottari M, Truong TB, Morosinotto T, Ahn TK, Fleming GR, et al (2011) Analysis of LhcSR3, a protein essential for feedback de-excitation in the green alga *Chlamydomonas reinhardtii*. PLoS Biol 9: e1000577 10.1371/journal.pbio.1000577 21267060 PMC3022525

[17] Miura K, Yamano T, Yoshioka S, Kohinata T, Inoue Y, Taniguchi F, et al (2004) Expression profiling-based identification of CO_2_-responsive genes regulated by CCM1 controlling a carbon-concentrating mechanism in *Chlamydomonas reinhardtii*. Plant Physiology 135: 1595–1607. 10.1104/pp.104.041400 15235119 PMC519074

[18] Zhang Z, Shrager J, Jain M, Chang C, Vallon O, Grossman AR (2004) Insights into the survival of *Chlamydomonas reinhardtii* during sulfur starvation based on microarray analysis of gene expression. Eukaryotic Cell 3: 1331–1348. 10.1128/EC.3.5.1331-1348.2004 15470261 PMC522608

[19] Naumann B, Busch A, Allmer J, Ostendorf E, Zeller M, Kirchhoff H, et al (2007) Comparative quantitative proteomics to investigate the remodeling of bioenergetic pathways under iron deficiency in *Chlamydomonas reinhardtii*. Proteomics 7: 3964–3979. 10.1002/pmic.200700407 17922516

[20] Ledford HK, Baroli I, Shin JW, Fischer BB, Eggen RIL, Niyogi KK (2004) Comparative profiling of lipid-soluble antioxidants and transcripts reveals two phases of photo-oxidative stress in a xanthophyll-deficient mutant of *Chlamydomonas reinhardtii*. Molecular Genetics and Genomics 272: 470–479. 10.1007/s00438-004-1078-5 15517390

[21] Schmollinger S, Muhlhaus T, Boyle NR, Blaby IK, Casero D, Mettler T, et al (2014) Nitrogen-sparing mechanisms in *Chlamydomonas* affect the transcriptome, the proteome, and photosynthetic metabolism. Plant Cell 26: 1410–1435. 10.1105/tpc.113.122523 24748044 PMC4036562

[22] Koziol AG, Borza T, Ishida K, Keeling P, Lee RW, Durnford DG (2007) Tracing the evolution of the light-harvesting antennae in chlorophyll *a/b*-containing organisms. Plant Physiol 143: 1802–1816. 10.1104/pp.106.092536 17307901 PMC1851817

[23] Niyogi KK, Truong TB (2014) Evolution of flexible non-photochemical quenching mechanisms that regulate light harvesting in oxygenic photosynthesis. Current Opinion in Plant Biology 16: 307–314. 10.1016/j.pbi.2013.03.011 23583332

[24] Castenholz RW, Garcia-Pichel F (2000) Cyanobacterial responses to UV-radiation In: Whitton BA, Potts M, editors. Ecology of cyanobacteria: their diversity in time and space. Dordrecht, the Netherlands: Kluwer Academic Publishers pp. 591–611.

[25] Kirk JTO (1994) Optics of UV-B radiation in natureal waters. Archiv fur Hydrobiologie Beiheft Ergebnisse der Limnologie 43: 1–16.

[26] Massana R (2011) Eukaryotic picoplankton in surface oceans. Annu Rev Microbiol 65: 91–110. 10.1146/annurev-micro-090110-102903 21639789

[27] Worden AZ, Follows MJ, Giovannoni SJ, Wilken S, Zimmerman AE, Keeling PJ (2015) Environmental science. Rethinking the marine carbon cycle: factoring in the multifarious lifestyles of microbes. Science 347: 1257594 10.1126/science.1257594 25678667

[28] Marin B, Melkonian M (2010) Molecular phylogeny and classification of the Mamiellophyceae class. nov. (Chlorophyta) based on sequence comparisons of the nuclear- and plastid-encoded rRNA operons. Protist 161: 304–336. 10.1016/j.protis.2009.10.002 20005168

[29] Not F, Latasa M, Marie D, Cariou T, Vaulot D, Simon N (2004) A single species, *Micromonas pusilla* (Prasinophyceae), dominates the eukaryotic picoplankton in the Western English Channel. Applied and Environmental Microbiology 70: 4064–4072. 10.1128/AEM.70.7.4064-4072.2004 15240284 PMC444783

[30] Simmons MP, Bachy C, Sudek S, van Baren MJ, Sudek L, Ares M Jr., et al (2015) Intron invasions trace algal speciation and reveal nearly identical Arctic and Antarctic *Micromonas* populations. Mol Biol Evol 32: 2219–2235. 10.1093/molbev/msv122 25998521 PMC4540971

[31] Treusch AH, Demir-Hilton E, Vergin KL, Worden AZ, Carlson CA, Donatz MG, et al (2012) Phytoplankton distribution patterns in the northwestern Sargasso Sea revealed by small subunit rRNA genes from plastids. The ISME Journal 6: 481–492. 10.1038/ismej.2011.117 21955994 PMC3280133

[32] Moreau H, Verhelst B, Couloux A, Derelle E, Rombauts S, Grimsley N, et al (2012) Gene functionalities and genome structure in *Bathycoccus prasinos* reflect cellular specializations at the base of the green lineage. Genome Biol 13: R74 10.1186/gb-2012-13-8-r74 22925495 PMC3491373

[33] Palenik B, Grimwood J, Aerts A, Rouze P, Salamov A, Putnam N, et al (2007) The tiny eukaryote *Ostreococcus* provides genomic insights into the paradox of plankton speciation. Proc Natl Acad Sci U S A 104: 7705–7710. 10.1073/pnas.0611046104 17460045 PMC1863510

[34] Worden AZ, Lee JH, Mock T, Rouze P, Simmons MP, Aerts AL, et al (2009) Green evolution and dynamic adaptations revealed by genomes of the marine picoeukaryotes *Micromonas*. Science 324: 268–272. 10.1126/science.1167222 19359590

[35] Six C, Worden AZ, Rodriguez F, Moreau H, Partensky F (2005) New insights into the nature and phylogeny of prasinophyte antenna proteins: *Ostreococcus tauri*, a case study. Mol Biol Evol 22: 2217–2230. 10.1093/molbev/msi220 16049197

[36] Slapeta J, Lopez-Garcia P, Moreira D (2006) Global dispersal and ancient cryptic species in the smallest marine eukaryotes. Molecular Biology and Evolution 23: 23–29. 10.1093/molbev/msj001 16120798

[37] Keller MD, Selvin RC, Claus W, Guillard RRL (1987) Media for the culturing of oceanic ultraplankton. Journal of Phycology 23: 633.

[38] Kurn N, Chen P, Heath JD, Kopf-Sill A, Stephens KM, Wang S (2005) Novel isothermal, linear nucleic acid amplification systems for highly multiplexed applications. Clin Chem 51: 1973–1981. 10.1373/clinchem.2005.053694 16123149

[39] Li H, Durbin R (2009) Fast and accurate short read alignment with Burrows-Wheeler transform. Bioinformatics 25: 1754–1760. 10.1093/bioinformatics/btp324 19451168 PMC2705234

[40] Langmead B, Trapnell C, Pop M, Salzberg SL (2009) Ultrafast and memory-efficient alignment of short DNA sequences to the human genome. Genome Biol 10: R25 10.1186/gb-2009-10-3-r25 19261174 PMC2690996

[41] R-Core-Team (2015) R: A language and environment for statistical computing. 3 ed. Vienna, Austria: R Foundation for Statistical Computing

[42] McDonald SM, Plant JN, Worden AZ (2010) The mixed lineage nature of nitrogen transport and assimilation in marine eukaryotic phytoplankton: a case study of *Micromonas*. Mol Biol Evol 27: 2268–2283. 10.1093/molbev/msq113 20457585 PMC2944026

[43] Livak KJ, Schmittgen TD (2001) Analysis of relative gene expression data using real-time quantitative PCR and the 2(-Delta Delta C(T)) Method. Methods 25: 402–408. 10.1006/meth.2001.1262 11846609

[44] Smith DR (2013) RNA-Seq data: a goldmine for organelle research. Brief Funct Genomics 12: 454–456. 10.1093/bfgp/els066 23334532

[45] Trapnell C, Roberts A, Goff L, Pertea G, Kim D, Kelley DR, et al (2012) Differential gene and transcript expression analysis of RNA-seq experiments with TopHat and Cufflinks. Nat Protoc 7: 562–578. 10.1038/nprot.2012.016 22383036 PMC3334321

[46] Terada Y, Aoki H, Tanaka T, Morikawa H, Ida S (1995) Cloning and nucleotide sequence of a leaf ferredoxin-nitrite reductase cDNA of rice. Biosci Biotechnol Biochem 59: 2183–2185. 8541663 10.1271/bbb.59.2183

[47] Foulon E, Not F, Jalabert F, Cariou T, Massana R, Simon N (2008) Ecological niche partitioning in the picoplanktonic green alga *Micromonas pusilla*: evidence from environmental surveys using phylogenetic probes. Environ Microbiol 10: 2433–2443. 10.1111/j.1462-2920.2008.01673.x 18537812

[48] Simmons MP, Sudek S, Monier A, Limardo AJ, Jimenez V, Perle CR, et al (2016) Abundance and biogeography of picoprasinophyte ecotypes and other phytoplankton in the Eastern North Pacific Ocean Applied and Environmental Microbiology 82: 1693–1705. 10.1128/AEM.02730-15 26729718 PMC4784031

[49] Rodriguez F, Derelle E, Guillou L, Le Gall F, Vaulot D, Moreau H (2005) Ecotype diversity in the marine picoeukaryote *Ostreococcus* (Chlorophyta, Prasinophyceae). Environmental Microbiology 7: 853–859. 10.1111/j.1462-2920.2005.00758.x 15892704

[50] Six C, Finkel ZV, Rodriguez F, Marie D, Partensky F, Campbell DA (2008) Contrasting photoacclimation costs in ecotypes of the marine eukaryotic picoplankter *Ostreococcus*. Limnology and Oceanography 53: 255–265.

[51] DuRand M, Green R, Sosik H, Olson R (2002) Diel variations in optical properties of *Micromonas pusilla* (Prasinophyceae). Journal of Phycology 38: 1132–1142.

[52] Demir-Hilton E, Sudek S, Cuvelier ML, Gentemann C, Zehr JP, Worden AZ (2011) Global distribution patterns of distinct clades of the photosynthetic picoeukaryote *Ostreococcus*. The ISME Journal 5: 1095–1107. 10.1038/ismej.2010.209 21289652 PMC3146286

[53] Iriarte A, Purdie DA (1993) Photosynthesis and growth response of the oceanic picoplankter *Pycnococcus provasolii* Guillard (clone a48-23) (Chlorophyta) to variations in irradiance, photoperiod and temperature J Exp Mar Biol Ecol 168: 239–251.

[54] Davis M, Fiehn O, Durnford DG (2013) Metabolic acclimation to excess light intensity in *Chlamydomonas reinhardtii*. Plant, Cell & Environment 36: 1391–1405. 10.1111/pce.12071 23346954

[55] Bonente G, Pippa S, Castellano S, Bassi R, Ballottari M (2012) Acclimation of *Chlamydomonas reinhardtii* to different growth irradiances. J Biol Chem 287: 5833–5847. 10.1074/jbc.M111.304279 22205699 PMC3285353

[56] Jacquet S, Partensky F, Lennon J-F, Vaulot D (2001) Diel patterns of growth and division in marine picoplankton in culture Journal of Phycology 37: 357–369.

[57] Duanmu D, Bachy C, Sudek S, Wong CH, Jimenez V, Rockwell NC, et al (2014) Marine algae and land plants share conserved phytochrome signaling systems. Proc Natl Acad Sci U S A 111: 15827–15832. 10.1073/pnas.1416751111 25267653 PMC4226090

[58] Farinas B, Mary C, Manes CLD, Bhaud Y, Peaucellier G, Moreau H (2006) Natural synchronisation for the study of cell division in the green unicellular alga *Ostreococcus tauri*. Plant Molecular Biology 60: 277–292. 10.1007/s11103-005-4066-1 16429264

[59] Durnford DG, Price JA, McKim SM, Sarchfield ML (2003) Light-harvesting complex gene expression is controlled by both transcriptional and post-transcriptional mechanisms during photoacclimation in *Chlamydomonas reinhardtii*. Physiologia Plantarum 118: 193–205.

[60] Baroli I, Gutman BL, Ledford HK, Shin JW, Chin BL, Havaux M, et al (2004) Photo-oxidative stress in a xanthophyll-deficient mutant of *Chlamydomonas*. J Biol Chem 279: 6337–6344. 10.1074/jbc.M312919200 14665619

[61] Teramoto H, Itoh T, Ono T (2004) High-intensity-light-dependent and transient expression of new genes encoding distant relatives of light-harvesting chlorophyll-a/b proteins in *Chlamydomonas reinhardtii*. Plant Cell Physiology 45: 1221–1232. 10.1093/pcp/pch157 15509845

[62] Geider RJ, MacIntyre HL, Kana TM (1998) A dynamic regulatory model of phytoplanktonic acclimation to light, nutrients, and temperature. Limnology and Oceanography 43: 679–694.

[63] Anning T, MacIntyre HL, Pratt SM, Sammes PJ, Gibb S, Geider RJ (2000) Photoacclimation in the marine diatom *Skeletonema costatum*. Limnology and Oceanography 45: 1807–1817.

[64] MacIntyre HL, Kana TM, Anning T, Geider RJ (2002) Photoacclimation of photosynthesis irradiance response curves and photosynthetic pigments in microalgae and cyanobacteria. Journal of Phycology 38: 17–38.

[65] Post AF, Dubinsky Z, Wyman K, Falkowski PG (1984) Kinetics of light-intensity adaptation in a marine planktonic diatom. Marine Biology 83: 231–238.

[66] Kataria S, Jajoo A, Guruprasad KN (2014) Impact of increasing Ultraviolet-B (UV-B) radiation on photosynthetic processes. J Photochem Photobiol B 137: 55–66. 10.1016/j.jphotobiol.2014.02.004 24725638

[67] (2012) Technical Bulletin LU-8160. internet: Q-Lab Corporation.

[68] Tilbrook K, Dubois M, Crocco CD, Yin R, Chappuis R, Allorent G, et al (2016) UV-B Perception and Acclimation in *Chlamydomonas reinhardtii*. Plant Cell 28: 966–983. 10.1105/tpc.15.00287 27020958 PMC4863380

[69] Flombaum P, Gallegos JL, Gordillo RA, Rincon J, Zabala LL, Jiao N, et al (2013) Present and future global distributions of the marine Cyanobacteria *Prochlorococcus* and *Synechococcus*. Proc Natl Acad Sci U S A 110: 9824–9829. 10.1073/pnas.1307701110 23703908 PMC3683724

[70] Moulager M, Monnier A, Jesson B, Bouvet R, Mosser J, Schwartz C, et al (2007) Light-dependent regulation of cell division in *Ostreococcus*: evidence for a major transcriptional input. Plant Physiol 144: 1360–1369. 10.1104/pp.107.096149 17535824 PMC1914124

[71] Monnier A, Liverani S, Bouvet R, Jesson B, Smith JQ, Mosser J, et al (2010) Orchestrated transcription of biological processes in the marine picoeukaryote *Ostreococcus* exposed to light/dark cycles. BMC Genomics 11: 192 10.1186/1471-2164-11-192 20307298 PMC2850359

[72] Cohen I, Knopf JA, Irihimovitch V, Shapira M (2005) A proposed mechanism for the inhibitory effects of oxidative stress on Rubisco assembly and its subunit expression. Plant Physiology 137: 738–746. 10.1104/pp.104.056341 15681660 PMC1065373

[73] Kucho K, Okamoto K, Tabata S, Fukuzawa H, Ishiura M (2005) Identification of novel clock-controlled genes by cDNA macroarray analysis in *Chlamydomonas reinhardtii*. Plant Molecular Biology 57: 889–906. 10.1007/s11103-005-3248-1 15952072

[74] Lee J, Herrin DL (2002) Assessing the relative importance of light and the circadian clock in controlling chloroplast translation in *Chlamydomonas reinhardtii*. Photosynth Res 72: 295–306. 10.1023/A:1019881306640 16228528

[75] Zinser ER, Lindell D, Johnson ZI, Futschik ME, Steglich C, Coleman ML, et al (2009) Choreography of the transcriptome, photophysiology, and cell cycle of a minimal photoautotroph, *Prochlorococcus*. PLoS ONE 4: e5135 10.1371/journal.pone.0005135 19352512 PMC2663038

[76] Ito H, Mutsuda M, Murayama Y, Tomita J, Hosokawa N, Terauchi K, et al (2009) Cyanobacterial daily life with Kai-based circadian and diurnal genome-wide transcriptional control in *Synechococcus elongatus*. Proc Natl Acad Sci U S A 106: 14168–14173. 10.1073/pnas.0902587106 19666549 PMC2729038

[77] Waltman PH, Guo J, Reistetter EN, Purvine S, Ansong CK, van Baren MJ, et al (2016) Identifying aspects of the post-transcriptional program governing the proteome of the green alga *Micromonas pusilla*. PLoS One 11: e0155839 10.1371/journal.pone.0155839 27434306 PMC4951065

[78] Neilson JA, Durnford DG (2010) Structural and functional diversification of the light-harvesting complexes in photosynthetic eukaryotes. Photosynth Res 106: 57–71. 10.1007/s11120-010-9576-2 20596891

[79] Swingley WD, Iwai M, Chen Y, Ozawa S, Takizawa K, Takahashi Y, et al (2010) Characterization of photosystem I antenna proteins in the prasinophyte *Ostreococcus tauri*. Biochim Biophys Acta 1797: 1458–1464. 10.1016/j.bbabio.2010.04.017 20457235

[80] Andersson B, Aro EM (2001) Photodamage and D1 protein turnover in photosystem II In: Andersson B, Aro EM, editors. Photosynthesis and Respiration-Regulation of Photosynthesis. Dordrecht, The Netherlands: Kluwer Academic Publishers pp. 377–393.

[81] Teramoto H, Nakamori A, Minagawa J, Ono TA (2002) Light-intensity-dependent expression of Lhc gene family encoding light-harvesting chlorophyll-a/b proteins of photosystem II in *Chlamydomonas reinhardtii*. Plant Physiol 130: 325–333. 10.1104/pp.004622 12226512 PMC166565

[82] McKim SM, Durnford DG (2006) Translational regulation of light-harvesting complex expression during photoacclimation to high-light in *Chlamydomonas reinhardtii*. Plant Physiol Biochem 44: 857–865. 10.1016/j.plaphy.2006.10.018 17097295

[83] Choquet Y, Stern DB, Wostrikoff K, Kuras R, Girard-Bascou J, Wollman FA (1998) Translation of cytochrome *f* is autoregulated through the 5 ' untranslated region of *petA* mRNA in *Chlamydomonas* chloroplasts. Proceedings of the National Academy of Sciences of the United States of America 95: 4380–4385. 9539745 10.1073/pnas.95.8.4380 PMC22497

[84] Kuras R, Wollman FA (1994) The assembly of cytochrome *b6/f* complexes: an approach using genetic transformation of the green alga *Chlamydomonas reinhardtii*. EMBO J 13: 1019–1027. 8131736 10.1002/j.1460-2075.1994.tb06350.x PMC394909

[85] Wostrikoff K, Girard-Bascou J, Wollman FA, Choquet Y (2004) Biogenesis of PSI involves a cascade of translational autoregulation in the chloroplast of *Chlamydomonas*. EMBO J 23: 2696–2705. 10.1038/sj.emboj.7600266 15192706 PMC449776

[86] Shapira M, Lers A, Heifetz PB, Irihimovitz V, Osmond CB, Gillham NW, et al (1997) Differential regulation of chloroplast gene expression in *Chlamydomonas reinhardtii* during photoacclimation: light stress transiently suppresses synthesis of the Rubisco LSU protein while enhancing synthesis of the PS II D1 protein. Plant Mol Biol 33: 1001–1011. 9154982 10.1023/a:1005814800641

[87] Yoshioka-Nishimura M (2016) Close relations between the PSII repair cycle and thylakoid membrane dynamics. Plant Cell Physiol. 10.1093/pcp/pcw050 27017619

[88] Chaturvedi R, Shyam R (2000) Degradation and de novo synthesis of D1 protein and *psbA* transcript levels in *Chlamydomonas reinhardtii* during UV-B inactivation of photosynthesis and its reactivation. Journal of Biosciences 25: 65–71. 10824200 10.1007/BF02985183

[89] Pinnola A, Cazzaniga S, Alboresi A, Nevo R, Levin-Zaidman S, Reich Z, et al (2015) Light-harvesting complex stress-related proteins catalyze excess energy dissipation in both photosystems of *Physcomitrella patens*. The Plant Cell 27: 3213–3227. 10.1105/tpc.15.00443 26508763 PMC4682295

[90] Teramoto H, Ishii A, Kimura Y, Hasegawa K, Nakazawa S, Nakamura T, et al (2006) Action spectrum for expression of the high intensity light-inducible Lhc-like gene Lhl4 in the green alga *Chlamydomonas reinhardtii*. Plant Cell Physiol 47: 419–425. 10.1093/pcp/pcj009 16418228

[91] Kimura M, Yamamoto YY, Seki M, Sakurai T, Sato M, Abe T, et al (2003) Identification of *Arabidopsis* genes regulated by high light-stress using cDNA microarray. Photochemistry and Photobiology 77: 226–233. 12785063 10.1562/0031-8655(2003)077<0226:ioagrb>2.0.co;2

[92] Andersson U, Heddad M, Adamska I (2003) Light stress-induced one-helix protein of the chlorophyll a/b-binding family associated with photosystem I. Plant Physiol 132: 811–820. 10.1104/pp.102.019281 12805611 PMC167021

[93] Jansson S, Andersson J, Jung Kim S, Jackowski G (2000) An *Arabidopsis thaliana* protein homologous to cyanobacterial high-light-inducible proteins. Plant Molecular Biology 42: 345–351. 10794534 10.1023/a:1006365213954

[94] He Q, Dolganov N, Bjorkman O, Grossman AR (2001) The high light-inducible polypeptides in *Synechocystis* PCC6803. Expression and function in high light. J Biol Chem 276: 306–314. 10.1074/jbc.M008686200 11024039

[95] Huang L, McCluskey MP, Ni H, LaRossa RA (2002) Global gene expression profiles of the cyanobacterium Synechocystis sp. strain PCC 6803 in response to irradiation with UV-B and white light. J Bacteriol 184: 6845–6858. 10.1128/JB.184.24.6845-6858.2002 12446635 PMC135463

[96] Inoue-Kashino N, Kashino Y, Takahashi Y (2011) Psb30 is a photosystem II reaction center subunit and is required for optimal growth in high light in *Chlamydomonas reinhardtii*. J Photochem Photobiol B 104: 220–228. 10.1016/j.jphotobiol.2011.01.024 21356599

[97] Kovács-Bogdán E, Soll J, Bölter B (2010) Protein import into chloroplasts: the Tic complex and its regulation. Biochim Biophys Acta 1803: 740–747. 10.1016/j.bbamcr.2010.01.015 20100520

[98] Kikuchi S, Bédard J, Hirano M, Hirabayashi Y, Oishi M, Imai M, et al (2013) Uncovering the protein translocon at the chloroplast inner envelope membrane. Science 339: 571–574. 10.1126/science.1229262 23372012

[99] Hirasawa M, Tripathy JN, Somasundaram R, Johnson MK, Bhalla M, Allen JP, et al (2009) The interaction of spinach nitrite reductase with ferredoxin: a site-directed mutation study. Mol Plant 2: 407–415. 10.1093/mp/ssn098 19825625 PMC2902899

[100] Setif P, Hirasawa M, Cassan N, Lagoutte B, Tripathy JN, Knaff DB (2009) New Insights into the Catalytic Cycle of Plant Nitrite Reductase. Electron Transfer Kinetics and Charge Storage. Biochemistry 48: 2828–2838. 10.1021/bi802096f 19226104

[101] Calderon RH, Garcia-Cerdan JG, Malnoe A, Cook R, Russell JJ, Gaw C, et al (2013) A Conserved Rubredoxin Is Necessary for Photosystem II Accumulation in Diverse Oxygenic Photoautotrophs. Journal of Biological Chemistry 288: 26688–26696. 10.1074/jbc.M113.487629 23900844 PMC3772215

[102] Einsle O (2011) Structure and function of formate-dependent cytochrome c nitrite reductase, NrfA. Methods Enzymol 496: 399–422. 10.1016/B978-0-12-386489-5.00016-6 21514473

