# Supplementary material for: Responses of the picoprasinophyte Micromonas commoda to light and ultraviolet stress
Source: PLoS One. 2017 Mar 9;12(3):e0172135. doi: 10.1371/journal.pone.0172135 (PMC5344333; doi:10.1371/journal.pone.0172135)
Supplement: S1 File — This file contains supplementary Fig A and Table A. (DOCX) [file pone.0172135.s001.docx]

**S1 File. Supporting Information.**

**Fig A.** **Expression changes for protein-encoding genes from the chloroplast genome and a subset of nuclear genes relative to T_0_.** HL and HL+UV data represent fold changes relative to controls at the same time point, while each control time point is compared to the preceding control time point. Only genes that met coverage criteria and that displayed significant changes (*p*<0.01) ≥1.5-fold across the biological triplicates in at least one time point, relative to T_0_ are shown. Note that all LHCs and LIL as well as HSP90.2 (XP_002507383) and NIRFU (XP_002507511) proteins have transit peptides targeting them to the chloroplast, as predicted using TargetP.


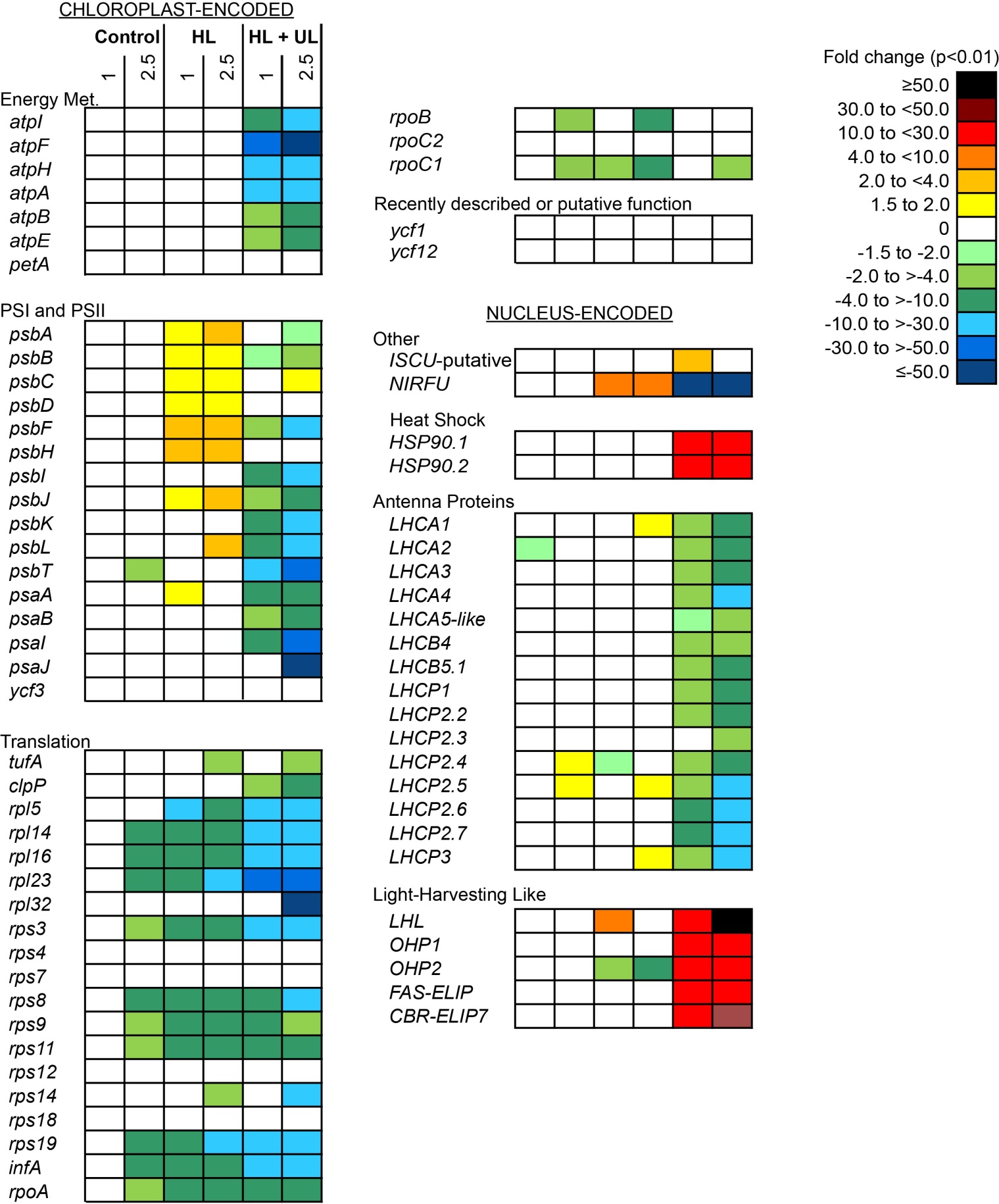


**Table A: BWA uniquely mapped reads per sample. Note the chloroplast genome is 72,585 bases long and the nuclear genome is 22 Mb. Thus the proportions of transcripts were highly skewed towards CP genes.**

| **Time point** | **biological replicate** | **# mapped to nuclear genome** | **# mapped to CP genome** |
| --- | --- | --- | --- |
| T0 | C0.I16_12 | 2,120,949 | 1,919,152 |
| T0 | C0.J16_13 | 2,252,388 | 1,885,757 |
| T0 | C0.L16_15 | 2,203,469 | 1,971,453 |
| T1 | C1.M16_16 | 2,686,026 | 2,642,775 |
| T1 | C1.N16_17 | 2,197,954 | 1,988,003 |
| T1 | C1.O16_18 | 3,553,756 | 3,569,868 |
| T2.5 | C2_5.Q16_20 | 1,328,004 | 1,123,828 |
| T2.5 | C2_5.R16_21 | 1,693,115 | 1,530,017 |
| T2.5 | C2_5.S16_22 | 2,399,808 | 2,706,806 |
| T1 | HL1.A15_01 | 2,540,286 | 2,192,859 |
| T1 | HL1.B15_02 | 2,505,698 | 2,115,917 |
| T1 | HL1.C15_03 | 2,672,677 | 2,214,111 |
| T2.5 | HL2_5.E15_05 | 1,819,966 | 1,557,370 |
| T2.5 | HL2_5.F15_06 | 1,573,432 | 1,494,440 |
| T2.5 | HL2_5.G15_07 | 2,325,083 | 2,117,910 |
| T0 | HL+UV0.U21_01 | 1,762,196 | 1,535,608 |
| T0 | HL+UV0.V21_02 | 2,312,611 | 2,284,689 |
| T0 | HL+UV0.W21_03 | 2,074,710 | 2,360,690 |
| T1 | HL+UV1.AA21_03 | 1,739,419 | 1,614,302 |
| T1 | HL+UV1.BB21_04 | 1,721,835 | 1,586,239 |
| T1 | HL+UV1.Z21_02 | 1,802,053 | 1,833,370 |
| T2.5 | HL+UV2_5.CC21_05 | 1,426,995 | 1,463,998 |
| T2.5 | HL+UV2_5.DD21_06 | 1,978,146 | 1,934,561 |
| T2.5 | HL+UV2_5.FF21_08 | 1,832,287 | 1,690,296 |
